# Is doxycycline post-exposure prophylaxis being utilised in Germany? Insights from an online survey among German men who have sex with men

**DOI:** 10.1007/s15010-024-02321-x

**Published:** 2024-07-23

**Authors:** Laura Wagner, Christoph Boesecke, Axel Baumgarten, Stefan Scholten, Sven Schellberg, Christian Hoffmann, Franz Audebert, Sebastian Noe, Johanna Erber, Marcel Lee, Julian Triebelhorn, Jochen Schneider, Christoph D. Spinner, Florian Voit

**Affiliations:** 1 TUM School of Medicine and Health, Department of Clinical Medicine, Clinical Department for Internal Medicine II, University Medical Center, Technical University of Munich, Munich, Germany; 2 University Hospital Bonn, Department of Internal Medicine I, Bonn, Germany; 3 German Centre for Infection Research (DZIF), Partner-Site Cologne-Bonn, Bonn, Germany; 4 Center for Infectiology, Berlin, Germany; 5 Private Practice, Hohenstaufenring, Cologne, Germany; 6 Novopraxis Berlin GbR, Berlin, Germany; 7 ICH Study Center, Hamburg, Germany; 8 Praxiszentrum Alte Mälzerei, Regensburg, Germany; 9 MVZ München am Goetheplatz, Munich, Germany

**Keywords:** Doxycycline, Human immunodeficiency virus (HIV), Post-exposure-prophylaxis (PEP), Pre-exposure-prophylaxis (PrEP), Sexually transmitted disease (STD), Sexually transmitted infection (STI)

## Abstract

**Purpose:**

Doxycycline post-exposure prophylaxis (Doxy-PEP) reduces the likelihood of Chlamydia and early syphilis by approximately two-thirds. Currently, data on the frequency of Doxy-PEP use in men who have sex with men (MSM) are limited. This study aimed to assess knowledge, attitude towards, and frequency of Doxy-PEP use among MSM in Germany.

**Methods:**

We conducted a national online survey in Germany from summer to fall 2023, recruiting MSM and transgender women. Participants were invited to complete the online survey through social media, online dating platforms, and print media advertisements with active recruitment and poster advertising in private practices, tertiary outpatient clinics, and MSM community events in Germany.

**Results:**

In total, 438 participants completed the survey and were included in the analysis, and 285 (65.1%) were living with the human immunodeficiency virus (HIV) or taking HIV-pre-exposure prophylaxis (PrEP). Overall, 170 participants (38.8%) had heard of Doxy-PEP, and 275 (62.8%) would consider taking it, but only 32 (7.3%) reported having ever taken Doxy-PEP. The most common reason for a negative attitude towards Doxy-PEP were apprehension about insufficient detailed information, and concerns about antibiotic resistance. Doxy-PEP users were more likely to be on HIV-PrEP, had a higher self-reported risk of bacterial sexually transmitted infections (STIs), and often had a history of bacterial STIs.

**Conclusion:**

The study demonstrated high awareness and strong interest in Doxy-PEP among MSM in Germany, most of whom were living with HIV or taking HIV-PrEP; however, the actual usage of Doxy-PEP remains low in the summer and fall of 2023.

**Supplementary Information:**

The online version contains supplementary material available at 10.1007/s15010-024-02321-x.

## Introduction

Sexually transmitted infections (STIs) are a significant public health burden, with over 1,000,000 new cases diagnosed worldwide every day [1, 2]. While pre-exposure prophylaxis (PrEP) and post-exposure prophylaxis (PEP) are effective in preventing human immunodeficiency virus (HIV) infection, there is growing interest in drug-based prophylaxis for bacterial STIs in the United States of America (USA), particularly among key populations such as men who have sex with men (MSM) [3].

PEP with doxycycline aims to prevent the development of bacterial STIs such as syphilis, Chlamydia, and gonorrhoea. In a single randomised study in the USA among MSM and transgender women using HIV-PrEP or living with HIV, taking 200 mg of doxycycline within 72 h of condomless sex, doxycycline post-exposure prophylaxis (Doxy-PEP) prevented approximately two-thirds of all syphilis, gonorrhoea, and Chlamydia infections. Doxy-PEP was used an average of four times a month, and no severe side effects were reported [4]. In a comparable study in Europe, similar results were reported for the prevention of syphilis and Chlamydia infections. However, Doxy-PEP does not effectively prevent gonorrhoea [5] because of the high tetracycline resistance rate of *Neisseria gonorrhoeae* in Europe [6]. In contrast, a study on Kenyan cisgender women showed no significant reduction in the incidence of bacterial STIs with Doxy-PEP, likely due to low adherence [7].

Therefore, Doxy-PEP, when taken regularly, has the potential to prevent bacterial STIs such as syphilis, Chlamydia, and gonorrhoea. However, its efficacy is contingent on local resistance of *Neisseria gonorrhoeae*.

Despite these potential benefits, the indiscriminate use of antibiotics requires careful consideration, especially in light of escalating antimicrobial resistance (AMR). The long-term effects of Doxy-PEP on AMR and the human microbiome remain unclear, necessitating further investigation into appropriate target groups for Doxy-PEP [8]. Nevertheless, Doxy-PEP has generated significant interest in MSM at high risk for bacterial STIs [9]. For instance, 1,093 (84%) of 1,301 users of a dating platform for MSM in the USA expressed a strong interest in Doxy-PEP use [9].

Currently, data on the frequency of Doxy-PEP use among MSM in Germany are limited. This study aimed to assess the knowledge, attitude towards and frequency of Doxy-PEP use in MSM living in Germany using an online survey.

## Methods

### Study design

A national online survey was conducted within MSM in Germany from 16 June to 13 October 2023. MSM and transgender women aged ≥ 18 years were included in this study. No exclusion criteria were applied.

The online survey was hosted on a Lamapoll© 2023 platform (Lamano GmbH & Co. KG, Berlin, Germany). Participants accessed the survey through the Uniform Resource Locator via QR codes following advertising on social media, online dating platforms, and print media. Active recruitment and poster advertising were also conducted in medical centres, such as private practices, tertiary outpatient clinics, and MSM community events in Germany. The recruitment site was not tracked and thus cannot be reported. The survey was viewed 4,024 times on an online dating platform for MSM.

The collected data included general demographic information such as country of origin and the highest level of education, as well as details on gender, sexual orientation, risky sexual behaviour (self-assessed risk of bacterial STIs and self-assessed risk for HIV on a scale from 1 = no risk to 5 = very high risk, and 6 = unsure, respectively), existence of a permanent relationship and, if yes, number and gender of the partner(s) in the current permanent relationship, date of last sex, number of sexual partners, frequency of condom use, and the reasons for condomless sex. Additionally, information on the number and type of previous bacterial STIs, HIV status, and, regarding people living with HIV (PLWH), time of infection, HIV PrEP use, and experiences with antibiotics used as PEP other than doxycycline was collected. Participants who reported knowledge of Doxy-PEP were queried about their experiences with Doxy-PEP, sources of obtaining it, events that occurred, bacterial STIs that were diagnosed after taking Doxy-PEP, and their willingness to retake it. No personally identifiable data were collected or stored.

### Ethics

This study was approved by the Ethics Committee of the Technical University of Munich, School of Medicine, University Hospital rechts der Isar, Munich, Germany (approval No. 2023-391-S-KK). This study was conducted in accordance with the principles of the Declaration of Helsinki. Before participating in the survey, participants provided consent for the privacy policy and further use of anonymised data after completing the online survey.

### Statistical analysis

Given the exploratory nature of this study, no formal power analysis was conducted. Statistical analyses were performed using SPSS Statistics version 29 (IBM, Armonk, New York, USA). Graphical design was performed using GraphPad Prism (version 5; GraphPad Software, Boston, Massachusetts, USA). Descriptive analyses were used to present qualitative data as absolute and relative frequencies (N, %). For quantitative variables, the number (N), median, interquartile range (IQR), minimum, and maximum, or in the case of normal distribution, the number (N), mean value, 95% confidence interval, minimum, and maximum were specified. Univariate analysis involved the chi-square test of independence for categorical variables and Mann–Whitney U test for continuous variables. No data imputation was performed.

## Results

### Demographical data

A total of 517 participants participated in the online survey, and 438 completed the questionnaire and were included in the analysis. The median self-reported age was 38 (IQR 31–47) years. Among the 438 participants, 435 (99.3%) were identified as male and two (0.5%) as female at birth. At the time of the survey, 431 participants (98.4%) were identified as male, two (0.5%) as trans men, and four (0.9%) as non-binary. Regarding the country of birth, 342 participants (78.1%) stated Germany, 48 participants (11.0%) stated the rest of Europe, and the remaining participants reported being born in Africa, North America, Australia and Central or South America. A master’s degree, diploma degree, or higher was held by 176 participants (40.2%), and the remaining participants reported having at least a secondary education certificate. Participants were asked about meningococcal B vaccination status, and 85 participants (19.4%) reported having received two or more vaccine doses. A total of 380 participants (86.8%) were identified as gay and 46 (10.5%) as bisexual. Seventy-six participants (17.4%) reported living with HIV, mostly for more than five years. Of the remaining participants, 209 (57.7%) reported receiving HIV-PrEP. Table 1 provides an overview of the baseline characteristics.

**Table 1:** Baseline characteristics of the total cohort and participants who had ever taken Doxy-PEP and those who had not

Characteristic	Total (*N* = 438)	Doxy-PEP (*N* = 32)	No Doxy-PEP (*N* = 406)
Age in years, median (IQR)	Not significant		
	38 (31–47)	42 (33–49.75)	38 (30.25–47)
Sex at birth, No. (%)	Not significant		
Male Female No information on sex	435 (99.3) 2 (0.5) 1 (0.2)	32 (100.0) 0 (0) 0 (0)	403 (99.3) 2 (0.5) 1 (0.2)
Current gender, No. (%)	Not significant		
Male Trans-man Non-binary Other	431 (98.4) 2 (0.5) 4 (0.9) 1 (0.2)	32 (100.0) 0 (0) 0 (0) 0 (0)	399 (98.3) 2 (0.5) 4 (1.0) 1 (0.2)
Country of birth, No. (%)	**P: 0.0002**		
Germany Rest of Europe North America Middle/South America Africa Australia	342 (78.1) 48 (11.0) 8 (1.8) 13 (3.0) 22 (5.0) 5 (1.1)	25 (78.1) 1 (3.1) 1 (3.1) 0 (0) 2 (6.3) 3 (9.4)	317 (78.1) 47 (11.6) 7 (1.7) 13 (3.2) 20 (4.9) 2 (0.5)
Length of stay in Germany, No. (%)	Not significant		
Less than 1 year 1–3 years 4–9 years 10–20 years More than 20 years	6 (6.4) 18 (19.2) 33 (35.1) 22 (23.4) 15 (16.0)	1 (14.3) 0 (0) 3 (42.9) 2 (28.6) 1 (14.3)	5 (5.7)) 18 (20.7) 30 (34.5) 20 (23.0) 14 (16.1)
Sexual orientation, No. (%)	Not significant		
Gay Bisexual Heterosexual Other	380 (86.8) 46 (10.5) 2 (0.5) 10 (2.3)	28 (87.5) 3 (9.4) 0 (0) 1 (3.1)	352 (86.7) 43 (10.6) 2 (0.5) 9 (2.2)
Highest level of education, No. (%)	Not significant		
No formal educational qualifications Secondary education certificate Apprenticeship certificate General university entrance qualification Bachelor’s degree University degree (Master’s, Diploma, etc.)	0 (0) 11 (2.5) 65 (14.8) 88 (20.1) 98 (22.4) 176 (40.2)	0 (0) 0 (0) 5 (15.6) 5 (15.6) 5 (15.6) 17 (53.1)	0 (0) 11 (2.7) 60 (14.8) 83 (20.4) 93 (22.9) 159 (39.2)
HIV status, No. (%)	Not significant		
PLWH HIV negative Not known	76 (17.4) 355 (81.1) 7 (1.6)	7 (21.9) 25 (78.1) 0 (0)	69 (17.0) 330 (81.3) 7 (1.7)
Initial diagnosis of HIV, No. (%)	Not significant		
In the previous 7 days In the previous 4 weeks In the previous 6 months In the previous 12 months In the previous 5 years More than 5 years ago Not known	1/76 (1.3) 1/76 (1.3) 2/76 (2.6) 1/76 (1.3) 17/76 (22.4) 53/76 (69.7) 1/76 (1.3)	0/7 (0) 0/7 (0) 0/7 (0) 0/7 (0) 0/7 (0) 7/7 (100.0) 0/7 (0)	1/69 (1.4) 1/69 (1.4) 2/69 (2.9) 1/69 (1.4) 17/69 (24.6) 46/69 (66.7) 1/69 (1.4)
PrEP uptake, No. (%)	**P: < 0.0001**		
Currently on PrEP Currently not on PrEP	209 /362 (57.7) 153/362 (42.3)	24/25 (96.0) 1/25 (4.0)	185/337 (54.9) 152/337 (45.1)
Meningococcal B vaccination, No. (%)	**P: < 0.0001**		
One dose of the vaccine Two or more doses of the vaccine Not known No vaccination	41 (9.4) 85 (19.4) 139 (31.7) 173 (39.5)	4 (12.5) 16 (50.0) 4 (12.5) 8 (25.0)	37 (9.1) 69 (17.0) 135 (33.3) 165 (40.6)

Doxy-PEP, doxycycline post-exposure-prophylaxis; N, total number of participants per group; IQR, interquartile range; No., number; HIV, human immunodeficiency virus; PLWH, people living with HIV; PrEP, pre-exposure-prophylaxis

*Note* Parameters are displayed as numbers (relative frequencies in percentages). No. represents the total number of participants in each column. The fraction x/y represents the number of positive responses (x) per participant who answered this question (y)

### Risky sexual behaviour

**Fig. 1:**
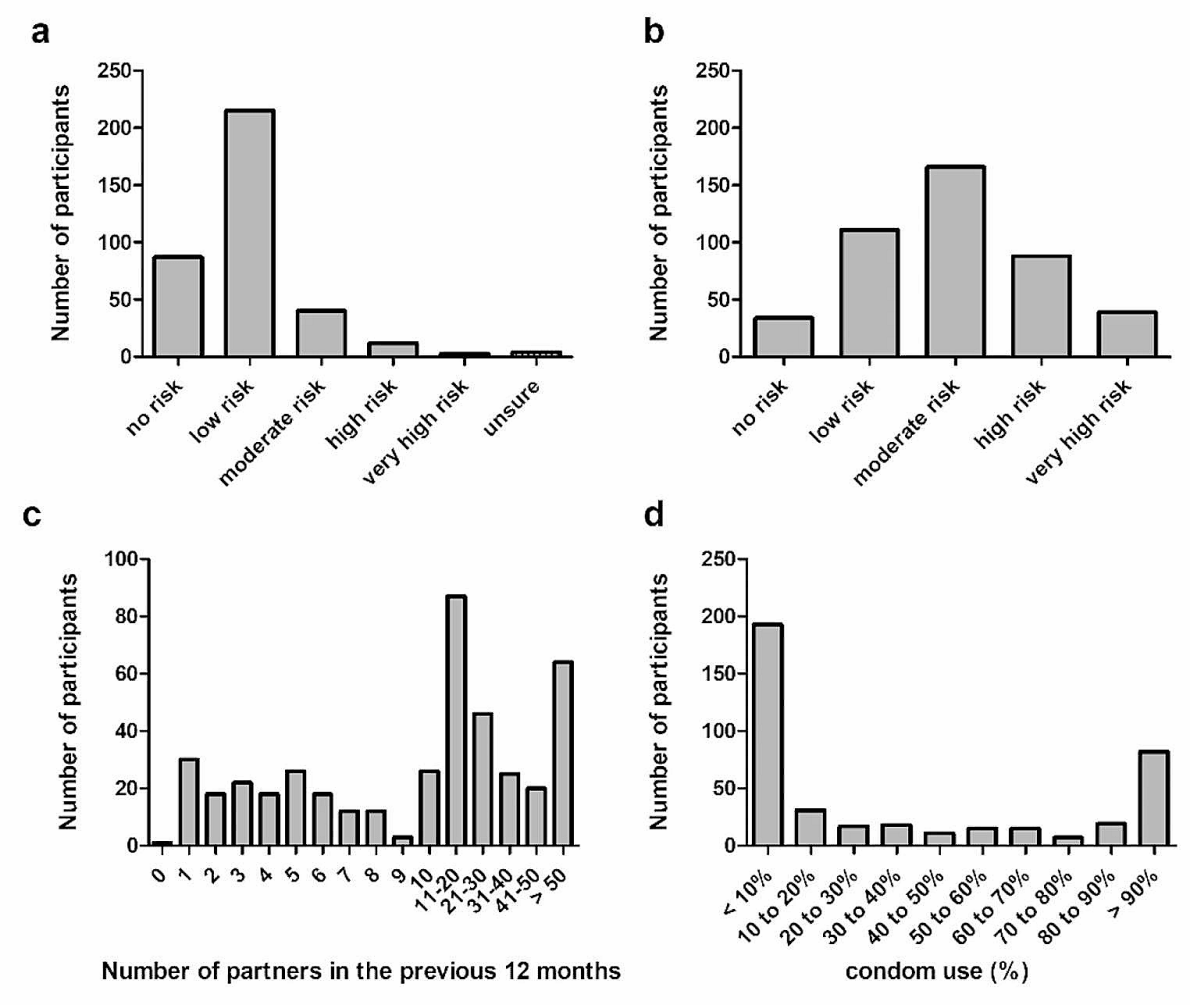
**a** : Perceived risk of acquiring HIV infection in the previous 12 months. Participants had to self-assess their risk on a scale of 1 (no risk) to 5 (very high-risk) and 6 (unsure). **b**: Perceived risk of acquiring a bacterial STI in the previous 12 months. Participants had to self-assess their risk on a scale of 1 (no risk) to 5 (very high-risk) and 6 (unsure). Refers only to bacterial STIs (not to HIV). **c**: Number of sexual partners in the previous 12 months. Refers to all male sexual contacts, only participants with last sexual contact < 1 year ago were included. **d**: Self-reported frequency of condom use. Refers only to participants with last self-reported sexual contact < 1 year. HIV, human immunodeficiency virus; STI, sexually transmitted infection

Regarding risky sexual behaviour, 87 (24.1%) HIV-negative participants described no relevant risk of HIV infection in the previous 12 months, 251 participants (70.7%) described a low to moderate risk, and only 15 participants (4.1%) described a high to very high risk of HIV infection (Fig. 1a). Concerning the risk of bacterial STI, 34 participants (7.8%) indicated no relevant risk, 277 participants (63.2%) indicated a low to moderate risk, and 127 participants (29%) indicated high to very high risk (Fig. 1b). Regarding relationship status, 194 participants (44.3%) had a permanent relationship with one man, 23 (5.3%) with more than one man, and 206 participants (47.0%) had no permanent relationship (Online Resource 1).

In total, 428 participants (97.7%) reported having sex with men in the previous year, and most participants reported having 11–30 or more than 50 male sexual partners (Fig. 1c). Of the 428 participants, 408 provided information on the frequency of condom use. Accordingly, 193 (47.3%) declared having used a condom in less than 10% and 82 (20.1%) declared having used a condom in more than 90% of all anal sexual intercourse cases in the previous year (Fig. 1d). One hundred ninety participants (43.4%) reported condomless sex in the previous nine days before participating in the survey. The most common reasons for condomless sex were that the participants preferred sex without a condom (184; 42.0%), the participant (195; 44.5%) or partner (151; 34.5%) was on HIV-PrEP, and the partner stated they did not have STIs or HIV infection (118; 26.9%).

Among the participants, 130 (29.7%) had been previously diagnosed with syphilis, whereas gonorrhoea and Chlamydia were reported by 207 (47.3%) and 192 (43.8%) participants. Additionally, there were less frequently reported cases of prior diagnoses of human papillomavirus, mycoplasma and/or *Ureaplasma spp.*, and herpes simplex virus infections. Detailed information on risky sexual behaviours is provided in Online Resources 1 and 2.

### Doxy-PEP

**Fig. 2:**
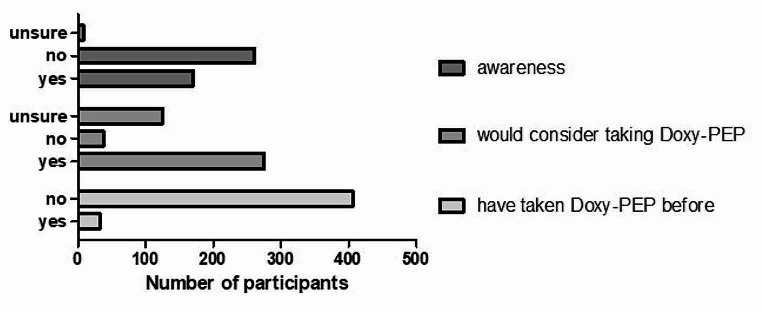
Doxy-PEP awareness. Prior to this study, all participants were asked about their knowledge of Doxy-PEP, willingness to take Doxy-PEP, and about having taken Doxy-PEP. Doxy-PEP, doxycycline post-exposure-prophylaxis

Concerning Doxy-PEP, 170 participants (38.8%) had knowledge of Doxy-PEP before the online survey, 260 (59.4%) did not have knowledge of it, and 8 (1.8%) were unsure (Fig. 2). The knowledge of Doxy-PEP was assessed in the three subgroups of (1) participants living with HIV (33/76 (43.4%)), (2) participants taking HIV-PrEP (80/209 (38.3%)), and (3) participants without HIV/without HIV-PrEP (57/153 (37.3%)). (Table 2)

**Table 2:** Knowledge of Doxy-PEP and willingness to take Doxy-PEP of the total cohort, of PLWH, of participants on HIV-PrEP, and of those living without HIV/not on HIV-PrEP

Characteristic	Total (*N* = 438)	PLWH (*N* = 76)	On HIV-PrEP (*N* = 209)	Living without HIV/not on HIV-PrEP (*N* = 153)
Had knowledge of Doxy-PEP, No. (%)				
Yes No Unsure	170 (38.8) 260 (59.4) 8 (1.8)	33 (43.4) 43 (56.6) 0 (0)	80 (38.3) 124 (59.3) 5 (2.4)	57 (37.3) 93 (60.8) 3 (2.0)
Had willingness to take Doxy-PEP, No. (%)				
Yes No Unsure	275 (62.8) 38 (8.7) 125 (28.5)	47 (61.8) 11 (14.5) 18 (23.7)	140 (67.0) 9 (4.3) 60 (28.7)	88 (57.5) 18 (11.8) 47 (30.7)

Doxy-PEP, doxycycline post-exposure prophylaxis; PLWH, participants living with the human immunodeficiency virus; HIV, human immunodeficiency virus; PrEP, pre-exposure prophylaxis; N, total number of participants per group; No., number

*Note* Parameters are displayed as numbers (relative frequencies in percentages)

There were no relevant differences in demographics and self-reported sexual risk behaviours between participants who had knowledge of Doxy-PEP and those who did not or were unsure. Participants with knowledge of Doxy-PEP reported more often having gonorrhoea or Chlamydia before the survey than participants without it (Online Resources 3 and 4).

Of all participants, 275 (62.8%) stated that they would consider taking Doxy-PEP, 38 (8.7%) stated that they would not consider taking it, and 125 (28.5%) were unsure. (Fig. 2) Willingness to take Doxy-PEP was highest among participants on HIV-PrEP (140/209 (67.0%)) compared to participants living with HIV (47/76 (61.8%)) and participants living without HIV/without HIV-PrEP (88/153 (57.5%)). (Table 2)

Participants who considered taking Doxy-PEP had a higher self-reported risk of bacterial STIs and preferred condomless sex more often than participants who did not consider taking it (Online Resource 5 and 6). Of all participants who did not consider taking it or who were unsure about it, 120 (73.6%) stated not being informed sufficiently, 66 (40.5%) had concerns about AMR, 44 (27.0%) did not want to take as many pills, 43 (26.4%) reported regular STI testing, 8 (4.9%) considered the effort of obtaining Doxy-PEP to be too great, 7 (4.3%) mentioned the prescription requirement, 6 (3.7%) indicated to be in a permanent relationship, 5 (3.1%) were concerned about side effects, 4 (2.5%) reported an allergy or intolerance to doxycycline, and 3 participants (1.8%) reported that their physician had advised against Doxy-PEP or that Doxy-PEP was too expensive (Table 3).

**Table 3:** Reasons for negative attitudes towards Doxy-PEP

Characteristic	Total (*N* = 163)
Insufficient information, No. (%)	120 (73.6)
Concerns about AMR, No. (%)	66 (40.5)
Too many pills, No. (%)	44 (27.0)
STI testing regularly	43 (26.4)
Effort of obtaining Doxy-PEP too great, No. (%)	8 (4.9)
Prescription requirement, No. (%)	7 (4.3)
Permanent relationship, No. (%)	6 (3.7)
Concerns about side effects, No. (%)	5 (3.1)
Allergy to doxycycline, No. (%)	4 (2.5)
Physicians advise, No. (%)	3 (1.8)
Costs are too high, No. (%)	3 (1.8)
Other reasons, No. (%)	4 (2.5)

Doxy-PEP, doxycycline post-exposure prophylaxis; No., number; AMR, antimicrobial resistance; STI, sexually transmitted infection

*Note* Parameters are displayed as numbers (relative frequencies in percentages). No. represents the total number of participants in each column. The total number of answers exceeded the total number of participants because multiple answer selections were possible

**Fig. 3:**
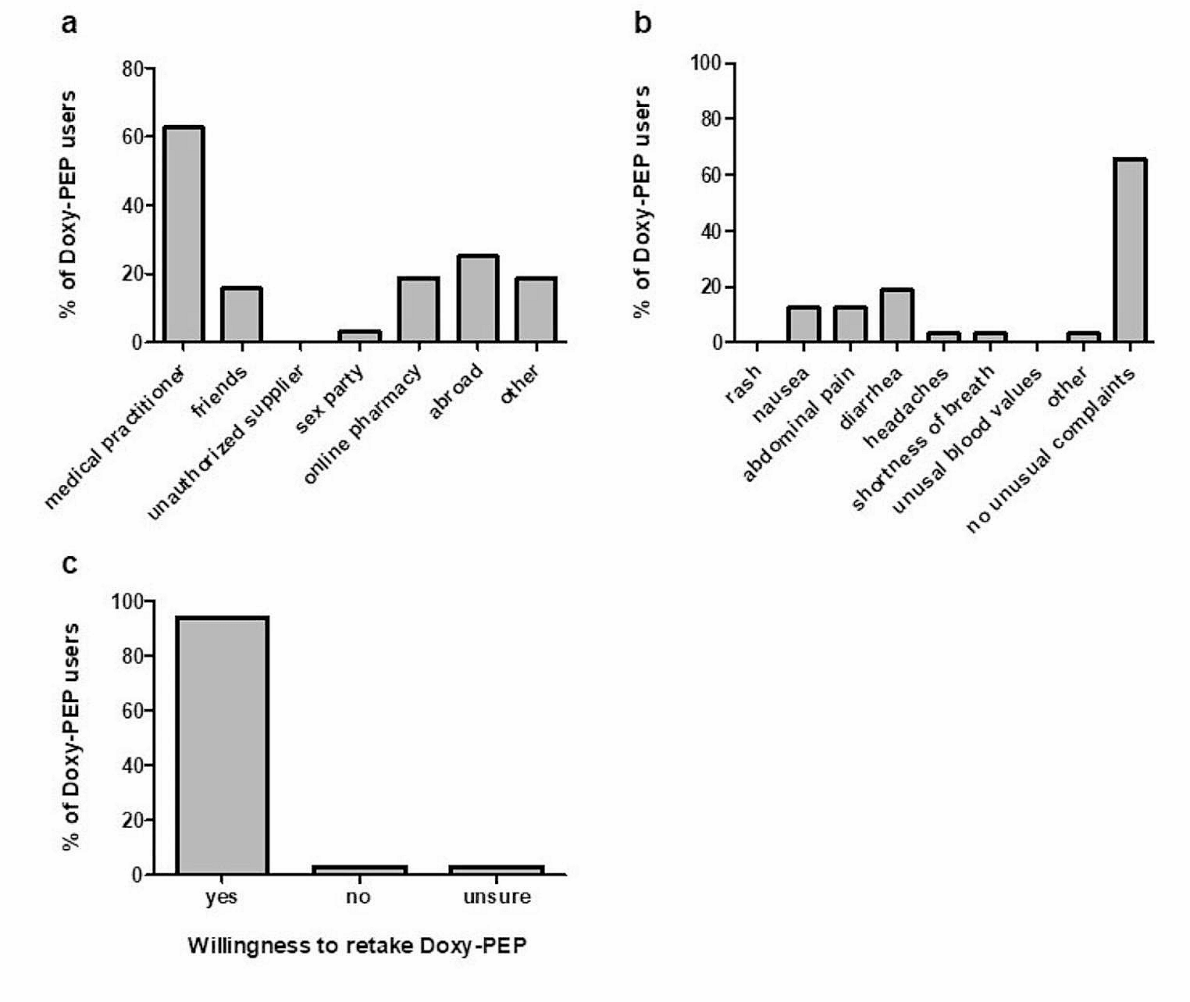
**a**: Sources of obtaining Doxy-PEP. The percentage refers to the total number of Doxy-PEP users (x-axis) for each possible answer (y-axis). The total percentage exceeds 100%, as selection of multiple answers was possible. **b**: Events that occurred after administering Doxy-PEP. The percentage refers to the total number of Doxy-PEP users (x-axis) for each possible answer (y-axis). The total percentage exceeds 100%, as selection of multiple answers was possible. **c**: Willingness to retake Doxy-PEP. Participants who had already taken Doxy-PEP prior to the survey were asked about their willingness to retake Doxy-PEP. Doxy-PEP, doxycycline post-exposure-prophylaxis

Thirty-two of the 438 participants (7.3%) confirmed the use of Doxy-PEP (Fig. 2). In most cases, Doxy-PEP was reported to be prescribed by a physician (20/32, 62.5%); less frequently, it was reported to be obtained abroad (8/32, 25%), from an online pharmacy (6/32; 18.8%), and friends or network (5/32; 15.6%) (Fig. 3a). Of the 32 participants, 21 did not report any events related to Doxy-PEP; diarrhoea was reported to occur in six cases (18.8%), and nausea and abdominal pain in four cases (12.5%) each (Fig. 3b). Thirty participants (93.8%) noted a positive experience and indicated a willingness to reuse Doxy-PEP. Only one participant was unsure and another opted against future use owing to concerns over antibiotic resistance (Fig. 3c). Overall, experiences with Doxy-PEP were predominately favourable, with only four participants (12.5%) rating it as an average, and four (12.5%) were unsure.

**Fig. 4:**
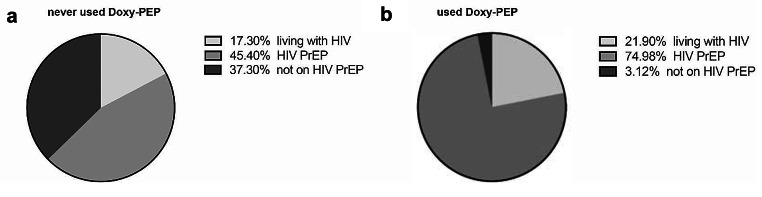
**a**: HIV status and HIV-PrEP use in participants not using Doxy-PEP. The percentage refers to all participants who did not use Doxy-PEP before the study. **b**: HIV status and HIV-PrEP use in Doxy-PEP users. The percentage refers to all participants who used Doxy-PEP prior to the study. HIV, human immunodeficiency virus; PrEP, pre-exposure-prophylaxis; Doxy-PEP, doxycycline post-exposure-prophylaxis

In a subgroup analysis of the 32 participants who had previously taken Doxy-PEP, seven participants (21.9%) reported living with HIV, a rate comparable to that of non-Doxy-PEP users, and all had been diagnosed over 5 years previously. The percentage of participants reporting the use of HIV PrEP was significantly higher in the Doxy-PEP group, with all but one participant confirming HIV-PrEP use (Table 1; Fig. 4a and b). Alongside the high usage of HIV-PrEP, 22 (88%) of HIV-negative participants rated their risk of HIV infection as non-existent or low, with only one participant (4%) indicating a high risk of HIV infection in the past 12 months. Regarding the risk of bacterial STIs, 20 (59.4%) disclosed a low to intermediate risk, and 12 (37.5%) disclosed a high to very high risk of acquiring a bacterial STI in the previous 12 months, a significantly higher proportion than that of non-Doxy-PEP users. (Online Resource 1)

All participants who reported having used Doxy-PEP acknowledged engaging in sexual activity with men at least once in the 6 months preceding the survey. Only one participant (3.1%) declared fewer than 10 partners, while 11 participants (34.4%) reported more than 50 partners in the previous 12 months. Self-reported use of condoms was infrequent, with 22 participants (68.8%) stating that they had used a condom in less than 10% of all male sexual encounters over the previous 12 months. Furthermore, 21 participants (65.7%) reported condomless sex in the nine days leading up to the survey. The most common reasons mirrored those observed in the overall cohort, with most participants or their individual partners confirming being on HIV-PrEP and/or expressing a preference for sex without a condom (Online Resource 1).

In the Doxy-PEP subgroup, STIs were significantly more commonly reported than in participants who had not reported taking Doxy-PEP before: 16 participants (50%) reported having been diagnosed with syphilis, 29 (90.6%) with gonorrhoea, and 24 (75.0%) with chlamydia. (Online Resource 1) Of all the participants who declared Doxy-PEP use, 25 (78.1%) mentioned having undergone testing for bacterial STIs within 3 months of Doxy-PEP use. Three participants were diagnosed with gonorrhoea, two with syphilis, and one with chlamydia.

Detailed information on the Doxy-PEP experience of the participants who reported having used Doxy-PEP is provided in Online Resource 7.

## Discussion

Doxy-PEP is a potential new approach for preventing bacterial STIs in key populations [10]. Although doxycycline is currently not approved for this indication, it is presumed to have high acceptability and is frequently used in the MSM community [9, 11–14]. To the best of our knowledge, the present study is the first comprehensive awareness study across Germany on the use of Doxy-PEP in MSM and transgender women.

The study population consisted mainly of sexually active MSM who reported living with HIV or taking HIV-PrEP. The baseline characteristics were comparable to those reported in studies on Doxy-PEP awareness in other countries, but with higher rates of PLWH and participants taking HIV-PrEP [9, 12, 14]. Despite employing a variety of recruitment methods, including social media, dating platforms, print media, and community events, our sample predominantly consists of MSM who likely have regular interactions with the German healthcare system. We did not specifically track the recruitment sites; however, the high proportion of participants with a history of HIV or those on HIV-PrEP suggests that a significant number may have been recruited from clinical centres focused on HIV treatment and prevention. This may influence their readiness to adopt new interventions like Doxy-PEP. Nevertheless, it is important to note that this bias might be somewhat mitigated by the fact that individuals with HIV or on HIV-PrEP are precisely the key populations for STI interventions. Additionally, the substantial traffic to our survey originating from an advertisement on an online dating platform indicates that a considerable segment of participants was also likely recruited via this channel. The high percentage of HIV-PrEP users might as well have contributed to the high rates of gonorrhoea and chlamydia in this study. HIV-PrEP use is known to be linked to increased numbers of sexual partners and a decreased use of condoms, which both enhance non-HIV STIs [15].

The observed level of awareness towards Doxy-PEP was high. There was also a largely positive self-reported attitude towards using Doxy-PEP, with the majority expressing willingness to consider using it, highlighting the potential demand and unmet need for alternative strategies to prevent bacterial STIs. This is consistent with the findings of other international awareness studies showing high rates of PEP acceptance of STIs [9, 12, 16, 17]. The most reported reason for a negative attitude was lack of knowledge, probably due to the off-label use status of Doxy-PEP in Germany at the time of the survey. This may also account for its infrequent prescription and low incidence of Doxy-PEP use. The demographics and social risk behaviour of participants who did not have knowledge of Doxy-PEP did not differ from those with knowledge of Doxy-PEP. As a result, there appears to be no majority that should be specifically targeted with information about Doxy-PEP. Participants with a high self-reported risk of bacterial STIs were up to take Doxy-PEP.

Overall, awareness of HIV or STIs was high, with only two participants reporting indifference to HIV or STIs. Condomless sex has been frequently reported, with one of the most common reasons being the use of HIV-PrEP. The survey indicated that taking Doxy-PEP was associated with self-reported HIV-PrEP use and a higher self-reported bacterial STI risk. However, this is probably due to the before mentioned bias towards MSM living with HIV or taking HIV-PrEP. In a US online survey, condomless sex and having had a bacterial STI were associated with a higher interest in Doxy-PEP use, but the use of HIV-PrEP or living with HIV was not associated with interest in Doxy-PEP when controlling for condom use and previously diagnosed bacterial STIs [9]. Nevertheless, this underscores the importance of combining strategies, such as drug prophylaxis (e.g., Doxy-PEP), with targeted education for the effective prevention of bacterial STIs, particularly among key populations. The European AIDS Clinical Society has proposed discussing Doxy-PEP use on a case-by-case basis in key populations in the recent 2023 guidelines [18]. Also, in 2023, the German STI Society likewise spoke out against the widespread introduction and daily use of the Doxy-PEP and recommended its use only in specific cases after individual risk assessment [19].

Overall, the reported experience with Doxy-PEP use was predominantly positive, with only two participants expressing reluctance or uncertainty about using it again due to concerns about AMR. AMR concerns were the second most common reason cited by those who had not used Doxy-PEP because of their negative perceptions of its use. The uncritical use of antibiotics is partly responsible for AMR development, making the untargeted prescription of these drugs increasingly undesirable [8]. In the ANRS IPERGAY trial by Molina et al. and the study by Luetkemeyer et al., the median amount of doxycycline used per participant in the Doxy-PEP group was 680 mg and 800 mg per month, respectively. However, it can be assumed that the use of antibiotics in the control group was also frequent, as approximately 30% of participants in the control group were diagnosed with bacterial STIs [4, 5]. In the study by Luetkemeyer et al., a higher resistance rate of *N. gonorrhoeae* was observed in the Doxy-PEP group. However, only a fraction of the patients with gonorrhoea underwent resistance testing. Similarly, Molina et al. found an increased rate of high-level resistant *N. gonorrhoeae* in individuals receiving Doxy-PEP, particularly against the backdrop of an overall higher resistance rate of *N. gonorrhoeae* in France. Considering these findings, further Doxy-PEP studies are required, with rigorous AMR monitoring, and regular STI and resistance testing should be performed when using Doxy-PEP [4, 5, 20, 21]. In this survey, gonorrhoea was the most frequently reported bacterial STI among Doxy-PEP users. Given that 91.2% of *N. gonorrhoeae* strains in Germany are currently resistant to tetracyclines, reduced efficacy of Doxy-PEP against *N. gonorrhoeae* in Germany can be anticipated [6, 22, 23].

An alternative approach for preventing infections with *N. gonorrhoeae* could be comprehensive vaccination of key populations against meningococcal serogroup B, as complete and partial vaccination series were 40% and 26% effective against gonorrhoea, respectively [24]. However, further studies are required to confirm these results [20, 25]. In this survey, approximately one-third of all participants and two-thirds of those who had taken Doxy-PEP reported partial or complete immunisation with meningococcal serogroup B vaccination. Although vaccination may provide less protection against gonorrhoea than Doxy-PEP, it could be associated with fewer risks of AMR development and offer long-term protection.

In addition to the aforementioned limitation of a bias towards MSM living with HIV or taking HIV-PrEP, a potential limitation of this study is the use of self-reported data, which can be affected by recall bias - a common issue in all non-representative surveys. This is particularly relevant concerning medical diagnoses. Furthermore, the survey did not collect information on where the participants resided in Germany, which may limit the generalisability of the results. Comparative studies should be conducted to assess the effectiveness, acceptability, and potential side effects of Doxy-PEP in comparison with other preventive measures, providing more comprehensive insights into the optimal strategies for STI prevention in key populations.

## Conclusion

This anonymous online survey indicated a high level of awareness and positive attitudes towards the use of Doxy-PEP among MSM in Germany, indicating both a potential demand and an unmet need for alternative preventive strategies against bacterial STIs. However, the survey primarily included responses from MSM who frequently interact with the healthcare system due to living with HIV or the use of HIV-PrEP. While this connection may influence their willingness to adopt new interventions such as Doxy-PEP, potentially limiting the generalizability of the findings, it is also critical to recognize that these individuals represent key populations for STI interventions.

## Electronic supplementary material

Below is the link to the electronic supplementary material.

Supplementary Material 1

Supplementary Material 2

Supplementary Material 3

Supplementary Material 4

Supplementary Material 5

Supplementary Material 6

Supplementary Material 7

## Data Availability

Data are available from the corresponding author upon request.
