# Supplementary material for: Is doxycycline post-exposure prophylaxis being utilised in Germany? Insights from an online survey among German men who have sex with men
Source: Infection. 2024 Jul 23;53(1):61–70. doi: 10.1007/s15010-024-02321-x (PMC11825561; doi:10.1007/s15010-024-02321-x)
Supplement: Supplementary file 1 — Supplementary Material 1 [file 15010_2024_2321_MOESM1_ESM.docx]

**Is doxycycline post-exposure prophylaxis being utilised in Germany? Insights from an online survey among German men who have sex with men**

Journal Name: *Infection*

Laura Wagner^1*^, Christoph Boesecke^2,3^, Axel Baumgarten^4^, Stefan Scholten^5^, Sven Schellberg^6^, Christian Hoffmann^7^, Franz Audebert^8^, Sebastian Noe^9^, Johanna Erber^1^, Marcel Lee^1^, Julian Triebelhorn^1^, Jochen Schneider^1^, Christoph D. Spinner^1^, Florian Voit^1^

^1^TUM School of Medicine and Health, Department of Clinical Medicine – Clinical Department for Internal Medicine II, University Medical Center, Technical University of Munich, Munich, Germany

^2^University Hospital Bonn, Department of Internal Medicine I, Bonn, Germany

^3^ German Centre for Infection Research (DZIF), partner-site Cologne-Bonn, Bonn, Germany

^4^ Center for Infectiology, Berlin, Germany

^5^ Private Practice, Hohenstaufenring, Cologne, Germany

^6^ Novopraxis Berlin GbR, Berlin, Germany

^7^ ICH Study Center, Hamburg, Germany

^8^ Praxiszentrum Alte Mälzerei, Regensburg, Germany

^9^ MVZ München am Goetheplatz, Munich, Germany

Corresponding author

Laura Wagner, MD

TUM School of Medicine and Health, Department of Clinical Medicine – Clinical Department for Internal Medicine II, University Medical Center, Technical University of Munich, Munich, Germany

Tel: +49 (89) 4140-9357

Fax: +49 (89) 4140-4808

Email: [laura.wagner@mri.tum.de](mailto:laura.wagner@mri.tum.de)

**Online Resource 2: Characteristics of risky sexual behaviour and STI history of PLWH, of participants on HIV-PrEP, and of those living without HIV/not on HIV-PrEP**

| Characteristic | PLWH  (N = 76) | On HIV-PrEP  (N = 209) | | Living without HIV/not on HIV-PrEP  (N = 153) |
| --- | --- | --- | --- | --- |
| Risk of HIV^a^, No. (%) |  | |  | |
| No risk  Low risk  Moderate risk  High risk  Very high risk  Not known |  | 55 (26.3)  124 (59.3)  20 (9.6)  5 (2.4)  3 (1.4)  2 (1.0) | | 32/152 (21.1)  91/152 (59.9)  20/152 (13.2)  7/152 (4.6)  0/152 (0)  2/152 (1.3) |
| Risk of bacterial STI^a^, No. (%) |  | |  | |
| No risk  Low risk  Moderate risk  High risk  Very high risk  Not known | 12 (15.8)  14 (18.4)  19 (25.0)  20 (26.3)  11 (14.5)  0 (0) | 5 (2.4)  29 (13.9)  93 (44.5)  55 (26.3)  27 (12.9)  0 (0) | | 17 (11.1)  68 (44.4)  54 (35.3)  13 (8.5)  1 (0.7)  0 (0) |
| Permanent relationship^b^, No. (%) |  | |  | |
| With one man  With more than one man  With one woman  With more than one woman  No permanent relationship  Other | 43 (56.6)  2 (2.6)  1 (1.3)  0 (0)  30 (39.5)  0 (0) | 91 (43.5)  16 (7.7)  2 (1.0)  0 (0)  101 (48.3)  1 (0.5) | | 60 (39.2)  5 (3.3)  11 (7.2)  1 (0.7)  75 (49.9)  3 (2.0) |
| Time of last male sexual contact^c^, No. (%) |  |  | |  |
| Never  Previous 24 hours  Previous 7 days  Previous 4 weeks  Previous 6 months  Previous 12 months  Previous 5 years  More than 5 years ago | 1 (1.3)  16 (21.1)  37 (48.7)  13 (17.1)  4 (5.3)  4 (5.3)  1 (1.3)  0 (0) | 0 (0)  63 (30.1)  103 (49.3)  36 (17.2)  4 (1.9)  1 (0.5)  1 (0.5)  1 (0.5) | | 1 (0.7)  26 (17.0)  59 (38.6)  43 (28.1)  17 (11.1)  2 (1.3)  4 (2.6)  1 (0.7) |
| Number of male sexual partners^d^, No. (%) |  |  | |  |
| 0  1  2  3  4  5  6  7  8  9  10  11–20  21–30  31–40  41–50  More than 50 | 0/74 (0)  8/74 (10.8)  5/74 (6.8)  2/74 (2.7)  6/74 (8.1)  2/74 (2.7)  4/74 (5.4)  1/74 (1.4)  1/74 (1.4)  0/74 (0)  3/74 (4.1)  10/74 (13.5)  11/74 (14.9)  4/74 (5.4)  4/74 (5.4)  13/74 (17.6) | 0/207 (0)  5/207 (2.4)  2/207 (1.0)  6/207 (2.9)  7/207 (3.4)  4/207 (1.9)  5/207 (2.4)  4/207 (1.9)  7/207 (3.4)  1/207 (0.5)  11/207 (5.3)  53/207 (25.6)  27/207 (13.0)  17/207 (8.2)  14/207 (6.8)  44/207 (21.3) | | 1/147 (0.7)  17/147 (11.6)  11/147 (7.5)  14/147 (9.5)  5/147 (3.4)  20/147 (13.6)  9/147 (6.1)  7/147 (4.8)  4/147 (2.7)  2/147 (1.4)  12/147 (8.2)  24/147 (16.3)  8/147 (5.4)  4/147 (2.7)  2/147 (1.4)  7/147 (4.8) |
| Frequency of condom use during male sex^e^, No. (%) |  |  | |  |
| <10%  10–20%  21–30%  31–40%  41–50%  51–60%  61–70%  71–80%  81–90%  >90% | 50/72 (69.4)  6/72 (8.3)  1/72(1.4)  1/72 (1.4)  3/72 (4.2)  1/72 (1.4)  2/72 (2.8)  0/72 (0)  2/72 (2.8)  6/72 (8.3) | 109/201 (54.2)  23/201 (11.4)  15/201 (7.5)  12/201 (6.0)  5/201 (2.5)  8/201 (4.0)  7/201 (3.5)  3/201 (1.5)  3/201 (1.5)  16/201 (8.0) | | 34/135 (25.2)  2/135 (1.5)  1/135 (0.7)  5/135 (3.7)  3/135 (2.2)  6/135 (4.4)  6/135 (4.4)  4/135 (3.0)  14/135 (10.4)  60/135 (44.4) |
| Time of last sexual contact with a woman^c^, No. (%) |  |  | |  |
| Never  Previous 24 hours  Previous 7 days  Previous 4 weeks  Previous 6 months  Previous 12 months  Previous 5 years  > 5 years | 42 (55.3)  0 (0)  0 (0)  0 (0)  0 (0)  0 (0)  3 (3.9)  31 (40.8) | 131 (62.7)  0 (0)  0 (0)  5 (2.4)  4 (1.9)  7 (3.3)  11 (5.3)  51 (24.4) | | 91 (59.9)  2 (1.3)  5 (3.3)  6 (3.9)  6 (3.9)  5 (3.3)  12 (7.8)  26 (17.0) |
| Number of female sexual partners^d^, No. (%) |  |  | |  |
| 1  2  3  4  5  6  7  8  9  10  11–20  21–30  31–40  41–50  More than 50 |  | 8/16 (50.0)  5/16 (31.3)  0/16 (0)  1/16 (6.3)  0/16 (0)  0/16 (0)  0/16 (0)  0/16 (0)  0/16 (0)  1/16 (6.3)  1/16 (6.3)  0/16 (0)  0/16 (0)  0/16 (0)  0/16 (0) | | 10/24 (41.7)  3/24 (12.5)  4/24 (16.7)  2/24 (8.3)  1/24 (4.2)  0/24 (0)  0/24 (0)  1/24 (4.2)  0/24 (0)  1/24 (4.2)  1/24 (4.2)  1/24 (4.2)  0/24 (0)  0/24 (0)  0/24 (0) |
| Frequency of condom use during female sex^e^, No. (%) |  |  | |  |
| <10%  10–20%  21–30%  31–40%  41–50%  51–60%  61–70%  71–80%  81–90%  >90% |  | 5/13 (38.5)  0/13 (0)  0/13 (0)  0/13 (0)  0/13 (0)  1/13 (7.7)  0/13 (0)  0/13 (0)  2/13 (15.4)  5/13 (38.5) | | 6/23 (26.1)  0/23 (0)  1/23 (4.3)  0/23 (0)  2/23 (8.7)  1/23 (4.3)  0/23 (0)  0/23 (0)  2/23 (8.7)  11/23 (47.8) |
| Reasons for condomless sex^b,e^, No. (%) |  |  | |  |
| Partner HIV-negative/STI free  Partner HIV-positive, undetectable viral load  HIV-positive, undetectable viral load  Partner refused condom use  Trusted partner  Partner on HIV PrEP  Participants on HIV PrEP  Indifferent to HIV/STI  Preference for condomless sex  Intoxicated/on substances  Condom broke or slipped off  No condom available  Permanent relationship  Other | 12 (15.8)  21 (27.6)  5 (6.6)  15 (19.7)  13 (17.1)  26 (34.2)  11 (14.5)  1 (1.3)  36 (47.4)  3 (3.9)  1 (1.3)  3 (3.9)  0 (0)  2 (2.6) | 44 (21.1)  16 (7.7)  0 (0)  31 (14.8)  39 (18.7)  93 (44.5)  176 (84.2)  1 (0.5)  111 (53.1)  13 (6.2)  4 (1.9)  10 (4.8)  1 (0.5)  4 (1.9) | | 62 (40.5)  5 (3.3)  0 (0)  19 (12.4)  50 (32.7)  32 (20.9)  8 (5.2)  0 (0)  37 (24.2)  9 (5.9)  2 (1.3)  13 (8.5)  6 (3.9)  9 (5.9) |
| Last condomless sex, No. (%) |  |  | |  |
| < 3 days  4–9 days  10–14 days  15 days–6 weeks  6 weeks–3 months  3 months–1 year  > 1 year  Never | 23 (30.3)  21 (27.6)  6 (7.9)  9 (11.8)  2 (2.6)  5 (6.6)  10 (13.2)  0 (0) | 70 (33.5)  47 (22.5)  25 (12.0)  25 (12.0)  9 (4.3)  9 (4.3)  16 (7.7)  8 (3.8) | | 13 (8.5)  16 (10.5)  12 (7.8)  16 (10.5)  21 (13.7)  24 (15.7)  27 (17.6)  24 (15.7) |
| History of syphilis, No. (%) |  |  | |  |
| Yes  No  Not known | 49 (64.5)  26 (34.2)  1 (1.3) | 64 (30.6)  143 (68.4)  2 (1.0) | | 17 (11.1)  134 (87.6)  2 (1.3) |
| Time of last syphilis, No. (%) |  |  | |  |
| Previous 24 hours  Previous 7 days  Previous 4 weeks  Previous 6 months  Previous 12 months  Previous 5 years  More than 5 years ago | 0/48 (0)  1/48 (2.1)  2/48 (4.2)  7/48 (14.6)  5/48 (10.4)  19/48 (39.6)  14/48 (29.2) | 0/64 (0)  2/64 (3.1)  2/61 (3.1)  7/64 (10.9)  15/64 (23.4)  28/64 (43.8)  10/64 (15.6) | | 0/17 (0)  0/17 (0)  0/17 (0)  1/17 (5.9)  3/17 (17.6)  7/17 (41.2)  6/17 (35.3) |
| History of gonorrhoea, No. (%) |  |  | |  |
| Yes  No  Not known | 48 (63.2)  25 (32.9)  3 (3.9) | 124 (59.3)  83 (39.7)  2 (1.0) | | 35 (22.9)  117 (76.5)  1 (0.7) |
| Time of last gonorrhoea, No. (%) |  |  | |  |
| Previous 24 hours  Previous 7 days  Previous 4 weeks  Previous 6 months  Previous 12 months  Previous 5 years  More than 5 years ago | 1/48 (2.1)  2/48 (4.2)  1/48 (2.1)  5/48 (10.4)  4/48 (8.3)  23/48 (47.9)  12/48 (25.0) | 1/124 (0.8)  4/124 (3.2)  14/124 (11.3)  29/124 (23.4)  26/124 (21.0)  36/124 (29.0)  14/124 (11.3) | | 1/35 (2.9)  0/35 (0)  1/35 (2.9)  5/35 (14.3)  5/35 (14.3)  15/35 (42.9)  8/35 (22.9) |
| History of chlamydia, No. (%) |  |  | |  |
| Yes  No  Not known | 39 (51.3)  35 (46.1)  2 (2.6) | 121 (57.9)  83 (39.7)  5 (2.4) | | 32 (20.9)  119 (77.8)  2 (1.3) |
| Time of last chlamydia, No. (%) |  |  | |  |
| Previous 24 hours  Previous 7 days  Previous 4 weeks  Previous 6 months  Previous 12 months  Previous 5 years  More than 5 years ago | 1/39 (2.6)  1/39 (2.6)  0/39 (0)  4/39 (10.3)  6/39 (15.4)  15/39 (38.5)  12/39 (30.8) | 0/121 (0)  1/121 (0.8)  10/121 (8.3)  227121 (18.2)  38/121 (31.4)  40/121 (33.1)  10/121 (8.3) | | 0/32 (0)  0/32 (0)  0/32 (0)  6/32 (18.8)  5/32 (15.6)  13/32 (40.6)  8/32 (25.0) |
| History of other bacterial STI, No. (%) |  |  | |  |
| Yes, details unknown  No  Not known  Mycoplasma/Ureaplasma  HSV  HPV | 0 (0)  56 (73.7)  13 (17.1)  3 (3.9)  2 (2.6)  3 (3.9) | 1 (0.5)  167 (79.9)  14 (6.7)  16 (7.7)  3 (1.4)  9 (4.3) | | 3 (2.0)  131 (85.6)  8 (5.2)  3 (2.0)  2 (1.3)  6 (3.9) |
| Time of other bacterial STI, No. (%) |  |  | |  |
| Previous 24 hours  Previous 7 days  Previous 4 weeks  Previous 6 months  Previous 12 months  Previous 5 years  More than 5 years ago | 0/9 (0)  0/9 (0)  1/9 (11.1)  1/9 (11.1)  1/9 (11.1)  5/9 (55.6)  1/9 (11.1) | 0/29 (0)  1/29 (3.4)  0/29 (0)  9/29 (31.0)  9/29 (31.0)  6/29 (20.7)  4/29 (13.8) | | 0/16 (0)  0/16 (0)  0/16 (0)  1/16 (6.3)  3/16 (18.8)  4/16 (25.0)  8/16 (50.0) |
| Have you taken other antibiotics for STI-PEP?, No. (%) |  |  | |  |
| No  Yes, substance unknown  Not known  Cefuroxime/Ceftriaxone  Azithromycin  Ciprofloxacin  Penicillin | 63 (82.9)  3 (3.9)  9 (11.8)  1 (1.3)  0 (0)  0 (0)  0 (0) | 180 (86.1)  10 (4.8)  15 (7.2)  1 (0.5)  2 (1.0)  0 (0)  1 (0.5) | | 139 (90.8)  1 (0,7)  10 (6.5)  1 (0.7)  1 (0.7)  1 (0.7)  0 (0) |

STI, sexually transmitted infection; PLWH, participants living with HIV; HIV-PrEP, human immunodeficiency virus-pre-exposure prophylaxis; N, total number of participants per group; No., number; HSV, herpes simplex virus; HPV, human papillomavirus; PEP, post-exposure prophylaxis.

Note: Parameters are displayed as numbers (relative frequencies in %). No. represents the total number of participants in each column. Fraction x/y represents the number of positive responses (x) per participant who answered this question (y). ^a^ Refers to the previous 12 months. ^b^ this was a multiple choice question, and the number of answers exceeds the total number of participants who answered this question ^c^ Time of last sexual contact refers to all sexual contacts. ^d^ Number of sexual partners refers to all sexual contacts; only participants with last sexual contact < 1 year ago were included. ^e^ Only participants with last sexual contact < 1 year ago were included.
