# Supplementary material for: Is doxycycline post-exposure prophylaxis being utilised in Germany? Insights from an online survey among German men who have sex with men
Source: Infection. 2024 Jul 23;53(1):61–70. doi: 10.1007/s15010-024-02321-x (PMC11825561; doi:10.1007/s15010-024-02321-x)
Supplement: Supplementary file 2 — Supplementary Material 2 [file 15010_2024_2321_MOESM2_ESM.docx]

**Is doxycycline post-exposure prophylaxis being utilised in Germany? Insights from an online survey among German men who have sex with men**

Journal Name: *Infection*

Laura Wagner^1*^, Christoph Boesecke^2,3^, Axel Baumgarten^4^, Stefan Scholten^5^, Sven Schellberg^6^, Christian Hoffmann^7^, Franz Audebert^8^, Sebastian Noe^9^, Johanna Erber^1^, Marcel Lee^1^, Julian Triebelhorn^1^, Jochen Schneider^1^, Christoph D. Spinner^1^, Florian Voit^1^

^1^TUM School of Medicine and Health, Department of Clinical Medicine – Clinical Department for Internal Medicine II, University Medical Center, Technical University of Munich, Munich, Germany

^2^University Hospital Bonn, Department of Internal Medicine I, Bonn, Germany

^3^ German Centre for Infection Research (DZIF), partner-site Cologne-Bonn, Bonn, Germany

^4^ Center for Infectiology, Berlin, Germany

^5^ Private Practice, Hohenstaufenring, Cologne, Germany

^6^ Novopraxis Berlin GbR, Berlin, Germany

^7^ ICH Study Center, Hamburg, Germany

^8^ Praxiszentrum Alte Mälzerei, Regensburg, Germany

^9^ MVZ München am Goetheplatz, Munich, Germany

Corresponding author

Laura Wagner, MD

TUM School of Medicine and Health, Department of Clinical Medicine – Clinical Department for Internal Medicine II, University Medical Center, Technical University of Munich, Munich, Germany

Tel: +49 (89) 4140-9357

Fax: +49 (89) 4140-4808

Email: laura.wagner@mri.tum.de

**Online Resource 3. Baseline characteristics of participants who had knowledge of Doxy-PEP and those who did not**

| Characteristic | Had knowledge of  Doxy-PEP  (N = 170) | Did not have knowledge of Doxy-PEP  (N = 268) |
| --- | --- | --- |
| Age in years, median (IQR) | Not significant | |
|  | 38.0 (29.0-46.0) | 38.0 (32.0-47.0) |
| Sex at birth, No. (%) | Not significant | |
| Male  Female  No information on sex | 169 (99.4)  1 (0.6)  0 (0) | 266 (99.3)  1 (0.4)  1 (0.4) |
| Current gender, No. (%) | Not significant | |
| Male  Trans-man  Non-binary  Other | 166 (97.6)  1 (0.6)  2 (1.2)  1 (0.6) | 265 (98.9)  1 (0.4)  2 (0.7)  0 (0) |
| Country of birth, No. (%) | Not significant | |
| Germany  Rest of Europe  North America  Middle/South America  Africa  Australia | 138 (81.2)  14 (8.2)  4 (2.4)  4 (2.4)  6 (3.5)  4 (2.4) | 204 (76.1)  34 (12.7)  4 (1.5)  9 (3.4)  16 (6.0)  1 (0.4) |
| Length of stay in Germany, No. (%) | Not significant |  |
| Less than 1 year  1–3 years  4–9 years  10–20 years  More than 20 years | 4/32 (12.5)  5/32 (15.6)  12/32 (37.5)  7/32 (21.9)  4/32 (12.5) | 2/204 (3.2)  13/204 (21.0)  21/204 (33.9)  15/204 (24.2)  11/204 (17.7) |
| Sexual orientation, No. (%) | Not significant | |
| Gay  Bisexual  Heterosexual  Other | 147 (86.5)  19 (11.2)  0 (0)  4 (2.4) | 233 (86.9)  27 (10.1)  2 (0.7)  6 (2.2) |
| Highest level of education, No. (%) | Not significant | |
| No formal educational qualifications  Secondary school certificate  Apprenticeship certificate  General university entrance qualification  Bachelor’s degree  University degree (Master’s, Diploma, etc.) | 0 (0)  2 (1.2)  25 (14.7)  34 (20.0)  34 (20.0)  75 (44.1) | 0 (0)  9 (3.4)  40 (14.9)  54 (20.1)  64 (23.9)  101 (37.7) |
| HIV status, No. (%) | Not significant | |
| PLWH  HIV negative  Not known | 33 (19.4)  135 (79.4)  2 (1.2) | 43 (16.0)  220 (82.1)  5 (1.9) |
| Initial diagnosis of HIV, No. (%) | Not significant |  |
| In the previous 7 days  In the previous 4 weeks  In the previous 6 months  In the previous 12 months  In the previous 5 years  More than 5 years ago  Not known | 1/33 (3.0)  0/33 (0)  0/33 (0)  1/33 (3.0)  5/33 (15.2)  26/33 (78.8)  0/33 (0) | 0/43 (0)  1/43 (2.3)  2/43 (4.7)  0/43 (0)  12/34 (27.9)  27/34 (62.8)  1/34 (2.3) |
| PrEP uptake, No. (%) | Not significant | |
| Currently on PrEP  Currently not on PrEP | 80/137 (58.4)  57/137 (41.6) | 129/225 (57.3)  96/225 (42.7) |
| Meningococcal B vaccination, No. (%) | Not significant |  |
| One dose of the vaccine  Two or more doses of the vaccine  Not known  No vaccination | 16 (9.4)  43 (25.3)  49 (28.8)  62 (36.5) | 25 (9.3)  42 (15.7)  90 (33.6)  111 (41.4) |

Doxy-PEP, doxycycline post-exposure-prophylaxis; N, total number of participants per group; IQR, interquartile range; No., number; HIV, human immunodeficiency virus; PLWH, people living with HIV; PrEP, pre-exposure prophylaxis.

Note: Parameters are displayed as numbers (relative frequencies in %). No. represents the total number of participants in each column. The fraction x/y represents the number of positive responses (x) per participant who answered this question (y).
