# Supplementary material for: Is doxycycline post-exposure prophylaxis being utilised in Germany? Insights from an online survey among German men who have sex with men
Source: Infection. 2024 Jul 23;53(1):61–70. doi: 10.1007/s15010-024-02321-x (PMC11825561; doi:10.1007/s15010-024-02321-x)
Supplement: Supplementary file 3 — Supplementary Material 3 [file 15010_2024_2321_MOESM3_ESM.docx]

**Is doxycycline post-exposure prophylaxis being utilised in Germany? Insights from an online survey among German men who have sex with men**

Journal Name: *Infection*

Laura Wagner^1*^, Christoph Boesecke^2,3^, Axel Baumgarten^4^, Stefan Scholten^5^, Sven Schellberg^6^, Christian Hoffmann^7^, Franz Audebert^8^, Sebastian Noe^9^, Johanna Erber^1^, Marcel Lee^1^, Julian Triebelhorn^1^, Jochen Schneider^1^, Christoph D. Spinner^1^, Florian Voit^1^

^1^TUM School of Medicine and Health, Department of Clinical Medicine – Clinical Department for Internal Medicine II, University Medical Center, Technical University of Munich, Munich, Germany

^2^University Hospital Bonn, Department of Internal Medicine I, Bonn, Germany

^3^ German Centre for Infection Research (DZIF), partner-site Cologne-Bonn, Bonn, Germany

^4^ Center for Infectiology, Berlin, Germany

^5^ Private Practice, Hohenstaufenring, Cologne, Germany

^6^ Novopraxis Berlin GbR, Berlin, Germany

^7^ ICH Study Center, Hamburg, Germany

^8^ Praxiszentrum Alte Mälzerei, Regensburg, Germany

^9^ MVZ München am Goetheplatz, Munich, Germany

Corresponding author

Laura Wagner, MD

TUM School of Medicine and Health, Department of Clinical Medicine – Clinical Department for Internal Medicine II, University Medical Center, Technical University of Munich, Munich, Germany

Tel: +49 (89) 4140-9357

Fax: +49 (89) 4140-4808

Email: laura.wagner@mri.tum.de

**Online Resource 5. Baseline characteristics of participants who would take Doxy-PEP and those who would not or were unsure.**

| Characteristic | Would take Doxy-PEP  (N = 275) | Would not take Doxy-PEP/unsure  (N = 163) |
| --- | --- | --- |
| Age in years, median (IQR) | Not significant | |
|  | 38.0 (30.0-47.0) | 40.0 (32.0-48.5) |
| Sex at birth, No. (%) | Not significant | |
| Male  Female  No information on sex | 272 (98.9)  2 (0.7)  1 (0.4) | 163 (100)  0 (0)  0 (0) |
| Current gender, No. (%) | Not significant | |
| Male  Trans-man  Non-binary  Other | 269 (97.8)  2 (0.7)  3 (1.1)  1 (0.4) | 162 (99.4)  0 (0)  1 (0.6)  0 (0) |
| Country of birth, No. (%) | Not significant | |
| Germany  Rest of Europe  North America  Middle/South America  Africa  Australia | 212 (77.1)  32 (11.6)  6 (2.2)  6 (2.2)  15 (5.5)  4 (1.5) | 130 (79.8)  16 (9.8)  2 (1.2)  7 (4.3)  7 (4.3)  1 (0.6) |
| Length of stay in Germany, No. (%) | Not significant |  |
| Less than 1 year  1–3 years  4–9 years  10–20 years  More than 20 years | 6/61 (9.8)  11/61 (18.0)  17/61 (27.9)  18/61 (29.5)  9/61 (14.8) | 0/33 (0)  7/33 (21.2)  16/33 (48.5)  4/33 (12.1)  6/33 (18.2) |
| Sexual orientation, No. (%) | Not significant | |
| Gay  Bisexual  Heterosexual  Other | 237 (86.2)  31 (11.3)  1 (0.4)  6 (2.2) | 143 (87.7)  15 (9.2)  1 (0.6)  4 (2.5) |
| Highest level of education, No. (%) | Not significant | |
| No formal educational qualifications  Secondary school certificate  Apprenticeship certificate  General university entrance qualification  Bachelor’s degree  University degree (Master’s, Diploma, etc.) | 0 (0)  4 (1.5)  34 (12.4)  56 (20.4)  63 (22.9)  118 (42.9) | 0 (0)  7 (4.3)  31 (19.0)  32 (19.6)  35 (21.5)  58 (35.6) |
| HIV status, No. (%) | Not significant | |
| PLWH  HIV negative  Not known | 47 (17.1)  225 (81.8)  3 (1.1) | 29 (17.8)  130 (79.8)  4 (2.5) |
| Initial diagnosis of HIV, No. (%) | Not significant |  |
| In the previous 7 days  In the previous 4 weeks  In the previous 6 months  In the previous 12 months  In the previous 5 years  More than 5 years ago  Not known | 1/47 (2.1)  1/47 (2.1)  2/47 (4.3)  1/47 (2.1)  7/47 (14.9)  35/47 (74.5)  0/47 (0) | 0/29 (0)  0/29 (0)  0/29 (0)  0/29 (0)  10/29 (34.5)  18/29 (62.1)  1/29 (3.4) |
| PrEP uptake, No. (%) | Not significant | |
| Currently on PrEP  Currently not on PrEP | 140/228 (61.4)  88/228 (38.6) | 69/134 (51.5)  65/134 (48.5) |
| Meningococcal B vaccination, No. (%) | Not significant |  |
| One dose of the vaccine  Two or more doses of the vaccine  Not known  No vaccination | 25 (9.1)  55 (20.0)  90 (32.7)  105 (38.2) | 16 (9.8)  30 (18.4)  49 (30.1)  68 (41.7) |

Doxy-PEP, doxycycline post-exposure-prophylaxis; N, total number of participants per group; IQR, interquartile range; No., number; HIV, human immunodeficiency virus; PLWH, people living with HIV; PrEP, pre-exposure prophylaxis.

Note: Parameters are displayed as number (relative frequency in %). No. represents the total number of participants in each column. The fraction x/y represents the number of positive responses (x) per participant who answered the question (y).
