# Supplementary material for: Is doxycycline post-exposure prophylaxis being utilised in Germany? Insights from an online survey among German men who have sex with men
Source: Infection. 2024 Jul 23;53(1):61–70. doi: 10.1007/s15010-024-02321-x (PMC11825561; doi:10.1007/s15010-024-02321-x)
Supplement: Supplementary file 4 — Supplementary Material 4 [file 15010_2024_2321_MOESM4_ESM.docx]

**Is doxycycline post-exposure prophylaxis being utilised in Germany? Insights from an online survey among German men who have sex with men**

Journal Name: *Infection*

Laura Wagner^1*^, Christoph Boesecke^2,3^, Axel Baumgarten^4^, Stefan Scholten^5^, Sven Schellberg^6^, Christian Hoffmann^7^, Franz Audebert^8^, Sebastian Noe^9^, Johanna Erber^1^, Marcel Lee^1^, Julian Triebelhorn^1^, Jochen Schneider^1^, Christoph D. Spinner^1^, Florian Voit^1^

^1^TUM School of Medicine and Health, Department of Clinical Medicine – Clinical Department for Internal Medicine II, University Medical Center, Technical University of Munich, Munich, Germany

^2^University Hospital Bonn, Department of Internal Medicine I, Bonn, Germany

^3^ German Centre for Infection Research (DZIF), partner-site Cologne-Bonn, Bonn, Germany

^4^ Center for Infectiology, Berlin, Germany

^5^ Private Practice, Hohenstaufenring, Cologne, Germany

^6^ Novopraxis Berlin GbR, Berlin, Germany

^7^ ICH Study Center, Hamburg, Germany

^8^ Praxiszentrum Alte Mälzerei, Regensburg, Germany

^9^ MVZ München am Goetheplatz, Munich, Germany

Corresponding author

Laura Wagner, MD

TUM School of Medicine and Health, Department of Clinical Medicine – Clinical Department for Internal Medicine II, University Medical Center, Technical University of Munich, Munich, Germany

Tel: +49 (89) 4140-9357

Fax: +49 (89) 4140-4808

Email: laura.wagner@mri.tum.de

**Online Resource 1. Characteristics of risky sexual behaviour and STI history of the total cohort and participants who had taken Doxy-PEP and those who had not**

| Characteristic | Total  (N = 438) | Doxy-PEP  (N = 32) | | No Doxy-PEP  (N = 406) |
| --- | --- | --- | --- | --- |
| Risk of HIV^a^, No. (%) | Not significant | |  | |
| No risk  Low risk  Moderate risk  High risk  Very high risk  Not known | 87/361 (24.1)  215/361 (59.6)  40/361 (11.1)  12/361 (3.3)  3/361 (0.8)  4/361 (1.1 | 9/25 (36.0)  13/25 (52.0)  2/25 (8.0)  0/25 (0)  1/25 (4.0)  0/25 (0) | | 78/336 (23.2)  202/336 (60.1)  38/336 (11.3)  12/336 (3.6)  2/336 (0.6)  4/336 (1.2) |
| Risk of bacterial STI^a^, No. (%) | **P: 0.0279** | |  | |
| No risk  Low risk  Moderate risk  High risk  Very high risk  Not known | 34 (7.8)  111 (25.3)  166 (37.9)  88 (20.1)  39 (8.9)  0 (0) | 1 (3.1)  1 (3.1)  18 (56.3)  7 (21.9)  5 (15.6)  0 (0) | | 33 (8.1)  110 (27.1)  148 (36.5)  81 (20.0)  34 (8.4)  0 (0) |
| Permanent relationship^b^, No. (%) | **P: 0.0140** | |  | |
| With one man  With more than one man  With one woman  With more than one woman  No permanent relationship  Other | 194 (44.3)  23 (5.3)  14 (3.2)  1 (0.2)  206 (47.0)  4 (0.9) | 14 (43.8)  6 (18.8)  0 (0)  0 (0)  12 (37.5)  0 (0) | | 180 (44.3)  17 (4.2)  14 (3.4)  1 (0.2)  194 (47.8)  4 (1.0) |
| Time of last male sexual contact^c^, No. (%) | Not significant |  | |  |
| Never  Previous 24 hours  Previous 7 days  Previous 4 weeks  Previous 6 months  Previous 12 months  Previous 5 years  More than 5 years ago | 2 (0.5)  105 (24.0)  199 (45.4)  92 (21.0)  25 (5.7)  7 (1.6)  6 (1.4)  2 (0.5) | 0 (0)  15 (46.9)  10 (31.3)  6 (18.8)  1 (3.1)  0 (0)  0 (0)  0 (0) | | 2 (0.5)  90 (22.2)  189 (46.6)  86 (21.2)  24 (5.9)  7 (1.7)  6 (1.5)  2 (0.5) |
| Number of male sexual partners^d^, No. (%) | Not significant |  | |  |
| 0  1  2  3  4  5  6  7  8  9  10  11–20  21–30  31–40  41–50  More than 50 | 1/428 (0.2)  30/428 (7.0)  18/428 (4.2)  22/428 (5.1)  18/428 (4.2)  26/428 (6.1)  18/428 (4.2)  12/428 (2.8)  12/428 (2.8)  3/428 (0.7)  26/428 (6.1)  87/428 (20.3)  46/428 (10.7)  25/428 (5.8)  20/428 (4.7)  64/428 (15.0) | 0 (0)  0 (0)  0 (0)  0 (0)  0 (0)  0 (0)  1 (3.1)  0 (0)  1 (3.1)  0 (0)  2 (6.3)  5 (15.6)  5 (15.6)  4 (12.5)  3 (9.4)  11 (34.4) | | 1/396 (0.3)  30/396 (7.6)  18/396 (4.5)  22/396 (5.6)  18/396 (4.5)  26/396 (6.6)  17/396 (4.3)  12/396 (3.0)  11/396 (2.8)  3/396 (0.8)  24/396 (6.1)  82/396 (20.7)  41/396 (10.4)  21/396 (5.3)  17/396 (4.3)  53/396 (13.4) |
| Frequency of condom use during male sex^e^, No. (%) | Not significant |  | |  |
| <10%  10–20%  21–30%  31–40%  41–50%  51–60%  61–70%  71–80%  81–90%  >90% | 193/408 (47.3)  31/408 (7.6)  17/408 (4.2)  18/408 (4.4)  11/408 (2.7)  15/408 (3.7)  15/408 (3.7)  7/408 (1.7)  19/408 (4.7)  82/408 (20.1) | 22 (68.8)  2 (6.3)  2 (6.3)  1 (3.1)  0 (0)  0 (0)  2 (6.3)  0 (0)  0 (0)  3 (9.4) | | 171/376 (45.5)  29/376 (7.7)  15/376 (4.0)  17/376 (4.5)  11/376 (2.9)  15/376 (4.0)  13/376 (3.5)  7/376 (1.9)  19/376 (5.1)  79/376 (21.0) |
| Time of last sexual contact with a woman^c^, No. (%) | Not significant |  | |  |
| Never  Previous 24 hours  Previous 7 days  Previous 4 weeks  Previous 6 months  Previous 12 months  Previous 5 years  > 5 years | 264 (60.3)  2 (0.5)  5 (1.1)  11 (2.5)  10 (2.3)  12 (2.7)  26 (5.9)  108 (24.7) | 23 (71.9)  0 (0)  0 (0)  2 (6.3)  1 (3.1)  0 (0)  4 (12.5)  2 (6.3) | | 241 (59.4)  2 (0.5)  5 (1.2)  9 (2.2)  9 (2.2)  12 (3.0)  22 (5.4)  106 (26.1) |
| Number of female sexual partners^d^, No. (%) | Not significant |  | |  |
| 1  2  3  4  5  6  7  8  9  10  11–20  21–30  31–40  41–50  More than 50 | 18/40 (45.0)  8/40 (20.0)  4/40 (10.0)  3/40 (7.5)  1/40 (2.5)  0/40 (0)  0/40 (0)  1/40 (2.5)  0/40 (0)  2/40 (5.0)  2/40 (5.0)  1/40 (2.5)  0/40 (0)  0/40 (0)  0/40 (0) | 0/3 (0)  2/3 (66.7)  0/3 (0)  0/3 (0)  0/3 (0)  0/3 (0)  0/3 (0)  0/3 (0)  0/3 (0)  0/3 (0)  1/3 (33.3)  0/3 (0)  0/3 (0)  0/3 (0)  0/3 (0) | | 18/37 (48.6)  6/37 (16.2)  4/37 (10.8)  3/37 (8.1)  1/37 (2.7)  0/37 (0)  0/37 (0)  1/37 (2.7)  0/37 (0)  2/37 (5.4)  1/37 (2.7)  1/37 (2.7)  0/37 (0)  0/37 (0)  0/37 (0) |
| Frequency of condom use during female sex^e^, No. (%) | Not significant |  | |  |
| <10%  10–20%  21–30%  31–40%  41–50%  51–60%  61–70%  71–80%  81–90%  >90% | 11/36 (30.6)  0/36 (0)  1/36 (2.8)  0/36 (0)  2/36 (5.6)  2/36 (5.6)  0/36 (0)  0/36 (0)  4/36 (11.1)  16/36 (44.4) | 1/3 (33.3)  0/3 (0)  0/3 (0)  0/3 (0)  0/3 (0)  0/3 (0)  0/3 (0)  0/3 (0)  1/3 (33.3)  1/3 (33.3) | | 10/33 (30.3)  0/33 (0)  1/33 (3.0)  0/33 (0)  2/33 (6.1)  2/33 (6.1)  0/33 (0)  0/33 (0)  3/33 (9.1)  15/33 (45.5) |
| Reasons for condomless sex^b,e^, No. (%) | Not significant |  | |  |
| Partner HIV-negative/STI free  Partner HIV-positive, undetectable viral load  HIV-positive, undetectable viral load  Partner refused condom use  Trusted partner  Partner on HIV-PrEP  Participants on HIV-PrEP  Indifferent to HIV/STI  Preference for condomless sex  Intoxicated/on substances  Condom broke or slipped off  No condom available  Permanent relationship  Other | 118 (26.9)  42 (9.6)  5 (1.1)  65 (14.8)  102 (23.3)  151 (34.5)  195 (44.5)  2 (0.5)  184 (42.0)  25 (5.7)  7 (1.6)  26 (5.9)  7 (1.6)  15 (3.4) | 3 (9.4)  4 (12.5)  1 (3.1)  5 (15.6)  2 (6.3)  15 (46.9)  22 (68.8)  0 (0)  19 (59.4)  2 (6.3)  1 (3.1)  2 (6.3)  0 (0)  2 (6.3) | | 115 (28.3)  38 (9.4)  4 (1.0)  60 (14.8)  100 (24.6)  136 (33.5)  173 (42.6)  2 (0.5)  165 (40.6)  23 (5.7)  6 (1.5)  24 (5.9)  7 (1.7)  13 (3.2) |
| Last condomless sex, No. (%) | Not significant |  | |  |
| < 3 days  4–9 days  10–14 days  15 days–6 weeks  6 weeks–3 months  3 months–1 year  > 1 year  Never | 106 (24.2)  84 (19.2)  43 (9.8)  50 (11.4)  32 (7.3)  38 (8.7)  53 (12.1)  32 (7.3) | 14 (43.8)  7 (21.9)  2 (6.3)  3 (9.4)  2 (6.3)  2 (6.3)  2 (6.3)  0 (0) | | 92 (22.7)  77 (19.0)  41 (10.1)  47 (11.6)  30 (7.4)  36 (8.9)  51 (12.6)  32 (7.9) |
| History of syphilis, No. (%) | **P: 0,0297** |  | |  |
| Yes  No  Not known | 130 (29.7)  303 (69.2)  5 (1.1) | 16 (50.0)  16 (50.0)  0 (0) | | 114 (28.1)  287 (70.7)  5 (1.2) |
| Time of last syphilis, No. (%) | Not significant |  | |  |
| Last 24 hours  Last 7 days  Last 4 weeks  Last 6 months  Last 12 months  Last 5 years  More than 5 years ago | 0/129 (0)  3/129 (2.3)  4/129 (3.1)  15/129 (11.6)  23/129 (17.8)  54/129 (41.9)  30/129 (23.3) | 0/16 (0)  1/16 (6.3)  0/16 (0)  1/16 (6.3)  4/16 (25.0)  6/16 (37.5)  4/16 (25.0) | | 0/113 (0)  2/113 (1.8)  4/113 (3.5)  14/113 (12.4)  19/113 (16.8)  48/113 (42.5)  26/113 (23.0) |
| History of gonorrhoea, No. (%) | **P: < 0.0001** |  | |  |
| Yes  No  Not known | 207 (47.3)  226 (51.4)  6 (1.4) | 29 (90.6)  3 (9.4)  0 (0) | | 178 (43.8)  222 (54.7)  6 (1.5) |
| Time of last gonorrhoea, No. (%) | Not significant |  | |  |
| Previous 24 hours  Previous 7 days  Previous 4 weeks  Previous 6 months  Previous 12 months  Previous 5 years  More than 5 years ago | 3/207 (1.4)  6/207 (2.9)  16/207 (7.7)  39/207 (18.8)  35/207 (16.9)  74/207 (35.7)  34/207 (16.4) | 0/29 (0)  1/29 (3.4)  5/29 (17.2)  7/29 (24.1)  6/29 (20.7)  5/29 (17.2)  5/29 (17.2) | | 3/178 (1.7)  5/178 (2.8)  11/178 (6.2)  32/178 (18.0)  29/178 (16.3)  69/178 (38.8)  29/178 (16.3) |
| History of chlamydia, No. (%) | **P: 0.0010** |  | |  |
| Yes  No  Not known | 192 (43.8)  237 (54.1)  9 (2.1) | 24 (75.0)  8 (25.0)  0 (0) | | 168 (41.1)  229 (56.4)  9 (2.2) |
| Time of last chlamydia, No. (%) | Not significant |  | |  |
| Previous 24 hours  Previous 7 days  Previous 4 weeks  Previous 6 months  Previous 12 months  Previous 5 years  More than 5 years ago | 1/192 (0.5)  2/192 (1.0)  10/192 (5.2)  32/192 (16.7)  49/192 (25.5)  68/192 (35.4)  30/192 (15.6) | 0/24 (0)  0/24 (0)  2/24 (8.3)  4/24 (16.7)  6/24 (25.0)  10/24 (41.7)  2/24 (8.3) | | 1/168 (0.6)  2/168 (1.2)  8/168 (4.8)  28/168 (16.7)  43/168 (25.6)  58/168 (34.5)  28/168 (16.7) |
| History of other bacterial STI, No. (%) | **P: < 0.0001** |  | |  |
| Yes, details unknown  No  Not known  Mycoplasma/Ureaplasma  HSV  HPV | 4 (0.9)  354 (80.8)  35 (8.0)  22 (5.0)  7 (1.6)  18 (4.1) | 0 (0)  19 (59.4)  4 (12.5)  6 (18.8)  4 (12.5)  1 (3.1) | | 4 (1.0)  335 (82.5)  31 (7.6)  16 (3.9)  3 (0.7)  17 (4.2) |
| Time of other bacterial STI, No. (%) | Not significant |  | |  |
| Previous 24 hours  Previous 7 days  Previous 4 weeks  Previous 6 months  Previous 12 months  Previous 5 years  More than 5 years ago | 0/54 (0)  1/54 (1.9)  1/54 (1.9)  11/54 (20.4)  13/54 (24.1)  15/54 (27.8)  13/54 (24.1) | 0/8 (0)  0/8 (0)  1/8 (12.5)  2/8 (25.0)  3/8 (37.5)  1/8 (12.5)  1/8 (12.5) | | 0/46 (0)  1/46 (2.2)  0/46 (0)  9 (19.6)  10/46 (21.7)  14/46 (30.4)  12/46 (26.1) |
| Have you taken other antibiotics for STI-PEP?, No. (%) | Not significant |  | |  |
| No  Yes, substance unknown  Not known  Cefuroxime/Ceftriaxone  Azithromycin  Ciprofloxacin  Penicillin | 382 (87.2)  14 (3.2)  34 (7.8)  3 (0.7)  3 (0.7)  1 (0.2)  1 (0.2) | 27 (84.4)  0 (0)  2 (6.3)  2 (6.3)  1 (3.1)  0 (0)  0 (0) | | 355 (87.4)  14 (3.4)  32 (7.9)  1 (0.2)  2 (0.5)  1 (0.2)  1 (0.2) |

STI, sexually transmitted infection; Doxy-PEP, doxycycline-post-exposure prophylaxis; N, total number of participants per group; HIV, human immunodeficiency virus; No., number; PrEP, pre-exposure prophylaxis; HSV, herpes simplex virus; HPV, human papillomavirus.

Note: Parameters are displayed as numbers (relative frequencies in %). No. represents the total number of participants in each column. Fraction x/y represents the number of positive responses (x) per participant who answered the question (y). ^a^ Refers to the previous 12 months. ^b^ this was a multiple choice question, and the number of answers exceeds the total number of participants who answered this question. ^c^ Time of last sexual contact refers to all sexual contacts. ^d^ Number of sexual partners refers to all sexual contacts; only participants with last sexual contact < 1 year ago were included. ^e^ Only participants with last sexual contact < 1 year ago were included.
