# Supplementary material for: Is doxycycline post-exposure prophylaxis being utilised in Germany? Insights from an online survey among German men who have sex with men
Source: Infection. 2024 Jul 23;53(1):61–70. doi: 10.1007/s15010-024-02321-x (PMC11825561; doi:10.1007/s15010-024-02321-x)
Supplement: Supplementary file 5 — Supplementary Material 5 [file 15010_2024_2321_MOESM5_ESM.docx]

**Is doxycycline post-exposure prophylaxis being utilised in Germany? Insights from an online survey among German men who have sex with men**

Journal Name: *Infection*

Laura Wagner^1*^, Christoph Boesecke^2,3^, Axel Baumgarten^4^, Stefan Scholten^5^, Sven Schellberg^6^, Christian Hoffmann^7^, Franz Audebert^8^, Sebastian Noe^9^, Johanna Erber^1^, Marcel Lee^1^, Julian Triebelhorn^1^, Jochen Schneider^1^, Christoph D. Spinner^1^, Florian Voit^1^

^1^TUM School of Medicine and Health, Department of Clinical Medicine – Clinical Department for Internal Medicine II, University Medical Center, Technical University of Munich, Munich, Germany

^2^University Hospital Bonn, Department of Internal Medicine I, Bonn, Germany

^3^ German Centre for Infection Research (DZIF), partner-site Cologne-Bonn, Bonn, Germany

^4^ Center for Infectiology, Berlin, Germany

^5^ Private Practice, Hohenstaufenring, Cologne, Germany

^6^ Novopraxis Berlin GbR, Berlin, Germany

^7^ ICH Study Center, Hamburg, Germany

^8^ Praxiszentrum Alte Mälzerei, Regensburg, Germany

^9^ MVZ München am Goetheplatz, Munich, Germany

Corresponding author

Laura Wagner, MD

TUM School of Medicine and Health, Department of Clinical Medicine – Clinical Department for Internal Medicine II, University Medical Center, Technical University of Munich, Munich, Germany

Tel: +49 (89) 4140-9357

Fax: +49 (89) 4140-4808

Email: laura.wagner@mri.tum.de

**Online Resource 7. Overview of Doxy-PEP use**

| Characteristic | Doxy-PEP  (N=32) |
| --- | --- |
| Where did you get Doxy-PEP?^a^, No. (%) |  |
| Physician  Friends  Dealer  Sex party  Online pharmacy  Abroad  Remaining stock of tablets  Other | 20 (62.5)  5 (15.6)  0 (0)  1 (3.1)  6 (18.8)  8 (25.0)  3 (9.4)  3 (9.4) |
| Would you take Doxy-PEP again?, No. (%) |  |
| Yes  Not  Not sure | 30 (93.8)  1 (3.1)  1 (3.1) |
| Events after taking Doxy-PEP^a^, No. (%) |  |
| Rash  Nausea  Abdominal pain  Diarrhoea  Headache  Shortness of breath  Unusual blood values  Other  No unusual complaints | 0 (0)  4 (12.5)  4 (12.5)  6 (18.8)  1 (3.1)  1 (3.1)  0 (0)  1 (3.1)  21 (65.6) |
| How would you rate Doxy-PEP?, No. (%) |  |
| Negative  Moderate  Positive  Not known | 0 (0)  4 (12.5)  24 (75.0)  4 (12.5) |
| STI testing 3 months after Doxy-PEP, No. (%) |  |
| Yes  No  Not known | 25 (78.1)  6 (18.8)  1 (3.1) |
| STI diagnosis 3 months after Doxy-PEP, No. (%) |  |
| Syphilis  Gonorrhoea  Chlamydia  Other  No STI | 2 (6.3)  3 (9.4)  1 (3.1)  0 (0)  19 (59.4) |

Doxy-PEP, doxycycline post-exposure prophylaxis; No., number; STI, sexually transmitted infection.

Note: Parameters are displayed as number (relative frequency in %). No. represents the total number of participants in each column. ^a^ The total number of answers exceeds the total number of participants because multiple selection of answers was possible.
