# Supplementary material for: Is doxycycline post-exposure prophylaxis being utilised in Germany? Insights from an online survey among German men who have sex with men
Source: Infection. 2024 Jul 23;53(1):61–70. doi: 10.1007/s15010-024-02321-x (PMC11825561; doi:10.1007/s15010-024-02321-x)
Supplement: Supplementary file 6 — Supplementary Material 6 [file 15010_2024_2321_MOESM6_ESM.docx]

**Is doxycycline post-exposure prophylaxis being utilised in Germany? Insights from an online survey among German men who have sex with men**

Journal Name: *Infection*

Laura Wagner^1*^, Christoph Boesecke^2,3^, Axel Baumgarten^4^, Stefan Scholten^5^, Sven Schellberg^6^, Christian Hoffmann^7^, Franz Audebert^8^, Sebastian Noe^9^, Johanna Erber^1^, Marcel Lee^1^, Julian Triebelhorn^1^, Jochen Schneider^1^, Christoph D. Spinner^1^, Florian Voit^1^

^1^TUM School of Medicine and Health, Department of Clinical Medicine – Clinical Department for Internal Medicine II, University Medical Center, Technical University of Munich, Munich, Germany

^2^University Hospital Bonn, Department of Internal Medicine I, Bonn, Germany

^3^ German Centre for Infection Research (DZIF), partner-site Cologne-Bonn, Bonn, Germany

^4^ Center for Infectiology, Berlin, Germany

^5^ Private Practice, Hohenstaufenring, Cologne, Germany

^6^ Novopraxis Berlin GbR, Berlin, Germany

^7^ ICH Study Center, Hamburg, Germany

^8^ Praxiszentrum Alte Mälzerei, Regensburg, Germany

^9^ MVZ München am Goetheplatz, Munich, Germany

Corresponding author

Laura Wagner, MD

TUM School of Medicine and Health, Department of Clinical Medicine – Clinical Department for Internal Medicine II, University Medical Center, Technical University of Munich, Munich, Germany

Tel: +49 (89) 4140-9357

Fax: +49 (89) 4140-4808

Email: laura.wagner@mri.tum.de

**Online Resource 4. Characteristics of risky sexual behaviour and STI history of participants who had knowledge of Doxy-PEP and those who did not**

| Characteristic | Had knowledge of Doxy-PEP  (N = 170) | Did not have knowledge of Doxy-PEP  (N = 268) |
| --- | --- | --- |
| Risk of HIV^a^, No. (%) | Not significant | |
| No risk  Low risk  Moderate risk  High risk  Very high risk  Not known | 30/137 (21.9)  84/137 (61.3)  17/137 (12.4)  4/137 (2.9)  1/137 (0.7)  1/137 (0.7) | 57/224 (25.4)  131/224 (58.5)  23/224 (10.3)  8/224 (3.6)  2/224 (0.9)  3/224 (1.3) |
| Risk of bacterial STI^a^, No. (%) | Not significant | |
| No risk  Low risk  Moderate risk  High risk  Very high risk  Not known | 13 (7.6)  37 (21.8)  67 (39.4)  34 (20.0)  19 (11.2)  0 (0) | 21 (7.8)  74 (27.6)  99 (36.9)  54 (20.1)  20 (7.5)  0 (0) |
| Permanent relationship^b^, No. (%) | Not significant | |
| With one man  With more than one man  With one woman  With more than one woman  No permanent relationship  Other | 71 (41.8)  11 (6.5)  2 (1.2)  1 (0.6)  84 (49.4)  3 (1.8) | 123 (45.9)  12 (4.5)  12 (4.5)  0 (0)  122 (45.5)  1 (0.4) |
| Time of last male sexual contact^c^, No. (%) | Not significant |  |
| Never  Previous 24 hours  Previous 7 days  Previous 4 weeks  Previous 6 months  Previous 12 months  Previous 5 years  More than 5 years ago | 1 (0.6)  44 (25.9)  80 (47.1)  31 (18.2)  10 (5.9)  3 (1.8)  1 (0.6)  0 (0) | 1 (0.4)  61 (22.8)  119 (44.4)  61 (22.8)  15 (5.6)  4 (1.5)  5 (1.9)  2 (0.7) |
| Number of male sexual partners^d^, No. (%) | Not significant |  |
| 0  1  2  3  4  5  6  7  8  9  10  11–20  21–30  31–40  41–50  More than 50 | 1/168 (0.6)  11/168 (6.5)  8/168 (4.8)  6/168 (3.6)  5/168 (3.0)  10/168 (6.0)  7/168 (4.2)  3/168 (1.8)  2/168 (1.2)  1/168 (0.6)  13/168 (7.7)  27/168 (16.1)  19/168 (11.3)  13/168 (7.7)  8/168 (4.8)  34/168 (20.2) | 0 (0)  19/260 (7.3)  10/260 (3.8)  16/260 (6.2)  13/260 (5.0)  16/260 (6.2)  11/260 (4.2)  9/260 (3.5)  10/260 (3.8)  2/260 (0.8)  13/260 (5.0)  60/260 (23.1)  27/260 (10.4)  12/260 (4.6)  12/260 (4.6)  30/260 (11.5) |
| Frequency of condom use during male sex^e^, No. (%) | Not significant |  |
| <10%  10–20%  21–30%  31–40%  41–50%  51–60%  61–70%  71–80%  81–90%  >90% | 80/158 (50.6)  11/158 (7.0)  6/158 (3.8)  7/158 (4.4)  5/158 (3.2)  5/158 (3.2)  7/158 (4.4)  3/158 (1.9)  6/158 (3.8)  28/158 (17.7) | 113/250 (45.2)  20/250 (8.0)  11/250 (4.4)  11/250 (4.4)  6/250 (2.4)  10/250 (4.0)  8/250 (3.2)  4/250 (1.6)  13/250 (5.2)  54/250 (21.6) |
| Time of last sexual contact with a woman^c^, No. (%) | Not significant |  |
| Never  Previous 24 hours  Previous 7 days  Previous 4 weeks  Previous 6 months  Previous 12 months  Previous 5 years  > 5 years | 104 (61.2)  1 (0.6)  1 (0.6)  6 (3.5)  4 (2.4)  5 (2.9)  13 (7.6)  36 (21.2) | 160 (59.7)  1 (0.4)  4 (1.5)  5 (1.9)  6 (2.2)  7 (2.6)  13 (4.9)  72 (26.9) |
| Reasons for condomless sex^b,e^, No. (%) | Not significant |  |
| Partner HIV-negative/STI free  Partner HIV-positive, undetectable viral load  HIV-positive, undetectable viral load  Partner refused condom use  Trusted partner  Partner on HIV-PrEP  Participants on HIV-PrEP  Indifferent to HIV/STI  Preference for condomless sex  Intoxicated/on substances  Condom broke or slipped off  No condom available  Permanent relationship  Other | 37 (21.8)  16 (9.4)  2 (1.2)  28 (16.5)  32 (18.8)  59 (34.7)  76 (44.7)  1 (0.6)  77 (45.3)  14 (8.2)  2 (1.2)  12 (7.1)  3 (1.8)  8 (4.7) | 81 (30.2)  26 (9.7)  3 (1.1)  37 (13.8)  70 (26.1)  92 (34.3)  119 (44.4)  1 (0.4)  107 (39.9)  11 (4.1)  5 (1.9)  14 (5.2)  4 (1.5)  7 (2.6) |
| Last condomless sex, No. (%) | Not significant |  |
| < 3 days  4–9 days  10–14 days  15 days–6 weeks  6 weeks–3 months  3 months–1 year  > 1 year  Never | 45 (26.5)  38 (22.4)  13 (7.6)  16 (9.4)  10 (5.9)  20 (11.8)  13 (7.6)  15 (8.8) | 61 (22.8)  46 (17.2)  30 (11.2)  34 (12.7)  22 (8.2)  18 (6.7)  40 (14.9)  17 (6.3) |
| History of syphilis, No. (%) | Not significant |  |
| Yes  No  Not known | 59 (34.7)  110 (64.7)  1 (0.6) | 71 (26.5)  193 (72.0)  4 (1.5) |
| History of gonorrhoea, No. (%) | **P: 0,0004** |  |
| Yes  No  Not known | 99 (58.2)  71 (41.8)  0 (0) | 108 (40.3)  154 (57.5)  6 (2.2) |
| History of chlamydia, No. (%) | **P: 0,0368** |  |
| Yes  No  Not known | 87 (51.2)  81 (47.6)  2 (1.2) | 105 (39.2)  156 (58.2)  7 (2.6) |

STI, sexually transmitted infection; Doxy-PEP, doxycycline post-exposure prophylaxis; N, total number of participants per group; HIV, human immunodeficiency virus; No., number; PrEP, pre-exposure prophylaxis.

Note: Parameters are displayed as number (relative frequency in %). No. represents the total number of participants in each column. The fraction x/y represents the number of positive responses (x) per participant who answered the question (y). ^a^ Refers to the previous 12 months. ^b^ The total number of answers exceeds the total number of participants because multiple selection of answers was possible. ^c^Time of last sexual contact refers to all sexual contacts. ^d^ Number of sexual partners refers to all sexual contacts; only participants with last sexual contact < 1 year ago were included. ^e^ Only participants with last sexual contact < 1 year ago were included.
