# Supplementary material for: Is doxycycline post-exposure prophylaxis being utilised in Germany? Insights from an online survey among German men who have sex with men
Source: Infection. 2024 Jul 23;53(1):61–70. doi: 10.1007/s15010-024-02321-x (PMC11825561; doi:10.1007/s15010-024-02321-x)
Supplement: Supplementary file 7 — Supplementary Material 7 [file 15010_2024_2321_MOESM7_ESM.docx]

**Is doxycycline post-exposure prophylaxis being utilised in Germany? Insights from an online survey among German men who have sex with men**

Journal Name: *Infection*

Laura Wagner^1*^, Christoph Boesecke^2,3^, Axel Baumgarten^4^, Stefan Scholten^5^, Sven Schellberg^6^, Christian Hoffmann^7^, Franz Audebert^8^, Sebastian Noe^9^, Johanna Erber^1^, Marcel Lee^1^, Julian Triebelhorn^1^, Jochen Schneider^1^, Christoph D. Spinner^1^, Florian Voit^1^

^1^TUM School of Medicine and Health, Department of Clinical Medicine – Clinical Department for Internal Medicine II, University Medical Center, Technical University of Munich, Munich, Germany

^2^University Hospital Bonn, Department of Internal Medicine I, Bonn, Germany

^3^ German Centre for Infection Research (DZIF), partner-site Cologne-Bonn, Bonn, Germany

^4^ Center for Infectiology, Berlin, Germany

^5^ Private Practice, Hohenstaufenring, Cologne, Germany

^6^ Novopraxis Berlin GbR, Berlin, Germany

^7^ ICH Study Center, Hamburg, Germany

^8^ Praxiszentrum Alte Mälzerei, Regensburg, Germany

^9^ MVZ München am Goetheplatz, Munich, Germany

Corresponding author

Laura Wagner, MD

TUM School of Medicine and Health, Department of Clinical Medicine – Clinical Department for Internal Medicine II, University Medical Center, Technical University of Munich, Munich, Germany

Tel: +49 (89) 4140-9357

Fax: +49 (89) 4140-4808

Email: laura.wagner@mri.tum.de

**Online Resource 6. Characteristics of risky sexual behaviour and STI history of participants who would take Doxy-PEP and those who would not or were unsure.**

| Characteristic | Would take Doxy-PEP  (N = 275) | Would not take Doxy-PEP/unsure  (N = 163) |
| --- | --- | --- |
| Risk of HIV^a^, No. (%) | Not significant | |
| No risk  Low risk  Moderate risk  High risk  Very high risk  Not known | 52/228 (22.8)  136/228 (59.6)  28/228 (12.3)  8/228 (3.5)  2/228 (0.9)  2/228 (0.9) | 35/133 (26.3)  79/133 (59.4)  12/133 (9.0)  4/133 (3.0)  1/133 (0.8)  2/133 (1.5) |
| Risk of bacterial STI^a^, No. (%) | **P: < 0.0001** | |
| No risk  Low risk  Moderate risk  High risk  Very high risk  Not known | 12 (4.4)  57 (20.7)  110 (40.0)  66 (24.0)  30 (10.9)  0 (0) | 22 (13.5)  54 (33.1)  56 (34.4)  22 (13.5)  9 (5.5)  0 (0) |
| Permanent relationship^b^, No. (%) | Not significant | |
| With one man  With more than one man  With one woman  With more than one woman  No permanent relationship  Other | 121 (44)  13 (4.7)  7 (2.5)  1 (0.4)  131 (47.6)  4 (1.5) | 73 (44.8)  10 (6.1)  7 (4.3)  0 (0)  75 (46.0)  0 (0) |
| Time of last male sexual contact^c^, No. (%) | Not significant |  |
| Never  Previous 24 hours  Previous 7 days  Previous 4 weeks  Previous 6 months  Previous 12 months  Previous 5 years  More than 5 years ago | 2 (0.7)  64 (23.3)  125 (45.5)  66 (24.0)  12 (4.4)  3 (1.1)  2 (0.7)  1 (0.4) | 0 (0)  41 (25.2)  74 (45.4)  26 (16.0)  13 (8.0)  4 (2.5)  4 (2.5)  1 (0.6) |
| Number of male sexual partners^d^, No. (%) | Not significant |  |
| 0  1  2  3  4  5  6  7  8  9  10  11–20  21–30  31–40  41–50  More than 50 | 1/270 (0.4)  10/270 (3.7)  7/270 (2.6)  16/270 (5.9)  11/270 (4.1)  18/270 (6.7)  14/270 (5.2)  7/270 (2.6)  8/270 (3.0)  2/270 (0.7)  14/270 (5.2)  57/270 (21.1)  31/270 (11.5)  17/270 (6.3)  12/270 (4.4.)  45/270 (16.7) | 0 (0)  20/158 (12.7)  11/158 (7.0)  6/158 (3.8)  7/158 (4.4)  8/158 (5.1)  4/158 (2.5)  5/158 (3.2)  4/158 (2.5)  1/158 (0.6)  12/158 (7.6)  30/158 (19.0)  15/158 (9.5)  8/158 (5.1)  8/158 (5.1)  19/158 (12.0) |
| Frequency of condom use during male sex^e^, No. (%) | Not significant |  |
| <10%  10–20%  21–30%  31–40%  41–50%  51–60%  61–70%  71–80%  81–90%  >90% | 118/257 (45.9)  21/257 (8.2)  9/257 (3.5)  15/257 (5.8)  8/257 (3.1)  8/257 (3.1)  12/257 (4.7)  5/257 (1.9)  13/257 (5.1)  48/257 (18.7) | 75/151 (49.7)  10/151 (6.6)  8/151 (5.3)  3/151 (2.0)  3/151 (2.0)  7/151 (4.6)  3/151 (2.0)  2/151 (1.3)  6/151 (4.0)  34/151 (22.5) |
| Time of last sexual contact with a woman^c^, No. (%) | Not significant |  |
| Never  Previous 24 hours  Previous 7 days  Previous 4 weeks  Previous 6 months  Previous 12 months  Previous 5 years  > 5 years | 168 (61.1)  2 (0.7)  4 (1.5)  7 (2.5)  9 (3.3)  6 (2.2)  18 (6.5)  61 (22.2) | 96 (58.9)  0 (0)  1 (0.6)  4 (2.5)  1 (0.6)  6 (3.7)  8 (4.9)  47 (28.8) |
| Reasons for condomless sex^b,e^, No. (%) | **P: 0,0208** |  |
| Partner HIV-negative/STI free  Partner HIV-positive, undetectable viral load  HIV-positive, undetectable viral load  Partner refused condom use  Trusted partner  Partner on HIV PrEP  Participants on HIV PrEP  Indifferent to HIV/STI  Preference for condomless sex  Intoxicated/on substances  Condom broke or slipped off  No condom available  Permanent relationship  Other | 67 (24.4)  24 (8.7)  2 (0.7)  43 (15.6)  57 (20.7)  107 (38.9)  133 (48.4)  1 (0.4)  128 (46.5)  17 (6.2)  2 (0.7)  19 (6.9)  2 (0.7)  12 (4.4) | 51 (31.1)  18 (11.0)  3 (1.8)  22 (13.5)  45 (27.6)  44 (27.0)  62 (38.0)  1 (0.6)  56 (34.4)  8 (4.9)  5 (3.1)  7 (4.3)  5 (3.1)  3 (1.8) |
| Last condomless sex, No. (%) | Not significant |  |
| < 3 days  4–9 days  10–14 days  15 days–6 weeks  6 weeks–3 months  3 months–1 year  > 1 year  Never | 65 (23.6)  56 (20.4)  27 (9.8)  32 (11.6)  22 (8.0)  24 (8.7)  31 (11.3)  18 (6.5) | 41 (25.2)  28 (17.2)  16 (9.8)  18 (11.0)  10 (6.1)  14 (8.6)  22 (13.5)  14 (8.6) |
| History of syphilis, No. (%) | Not significant |  |
| Yes  No  Not known | 91 (33.1)  189 (65.5)  4 (1.5) | 39 (23.9)  123 (75.5)  1 (0.6) |
| History of gonorrhoea, No. (%) | **P: 0,0605** |  |
| Yes  No  Not known | 140 (50.9)  133 (48.4)  2 (0.7) | 67 (41.1)  92 (56.4)  4 (2.5) |
| History of chlamydia, No. (%) | Not significant |  |
| Yes  No  Not known | 126 (45.8)  143 (52.0)  6 (2.2) | 66 (40.5)  94 (57.7)  3 (1.8) |

STI, sexually transmitted infection; Doxy-PEP, doxycycline post-exposure prophylaxis; N, total number of participants per group; HIV, human immunodeficiency virus; No., number; PrEP, pre-exposure prophylaxis.

Note: Parameters are displayed as numbers (relative frequencies in %). No. represents the total number of participants in each column. The fraction x/y represents the number of positive responses (x) per participant who answered the question (y). ^a^ Refers to the previous 12 months. ^b^ The total number of answers exceeds the total number of participants because multiple selection of answers was possible. ^c^ Time of last sexual contact refers to all sexual contacts. ^d^ Number of sexual partners refers to all sexual contacts; only participants with last sexual contact < 1 year ago were included. ^e^ Only participants with last sexual contact < 1 year ago were included.
